# Supplementary material for: Unraveling autophagic imbalances and therapeutic insights in Mecp2-deficient models
Source: EMBO Mol Med. 2024 Oct 14;16(11):2795–826. doi: 10.1038/s44321-024-00151-w (PMC11555085; doi:10.1038/s44321-024-00151-w)

Table of content of the Appendix

- Appendix Table S1…………………………………………………………………2
- Appendix Table S2…………………………………………………………………3
- Appendix Table S3…………………………………………………………………4
- Appendix Table S4…………………………………………………………………5
- Appendix Figure S1……………………………………………………………...6
- Appendix Figure S2 ……………………………………………………………..7
- Appendix Figure S3……………………………………………………………...8

Appendix Table S1. Significantly enriched KEGG terms of up-regulated genes in WT neurons upon trehalose treatment

KEGG terms	*P-adj*	*P-value*	Genes
Steroid biosynthesis	4,17E-09	2,05E-11	SQLE,NSDHL,SC5D,MSMO1,DHCR7,HSD17B7,LSS,TM7SF2
Lysosome	1,21E-03	1,19E-05	ARSA,CD164,CD63,ASAH1,NPC2,LAMP2,CTSZ,CTNS,MAN2B1
Fatty acid biosynthesis	2,58E-03	3,79E-05	FASN,ACSL4,ACSL3,ACACA
Ferroptosis	3,58E-03	8,17E-05	TFRC,HMOX1,ACSL4,ACSL3,SAT1
Terpenoid backbone biosynthesis	3,58E-03	8,76E-05	IDI1,MVK,MVD,ACAT2
Fatty acid degradation	0,037703658	1,24E-03	ACADL,ACSL4,ACSL3,ACAT2
PPAR signaling pathway	0,037703658	0,001312139	FADS2,ACADL,ACSL4,PLIN2,ACSL3
Gastric acid secretion	0,037703658	0,001478575	KCNJ10,KCNJ16,SLC4A2,EZR,GNAI2
Amino sugar and nucleotide sugar metabolism	0,04027664	0,001866425	GNPDA1,UAP1L1,HK2,GALK1
Sphingolipid signaling pathway	0,04027664	0,002000248	GNA13,SGPL1,ASAH1,S1PR1,TNFRSF1A,GNAI2

Appendix Table S2. Significantly enriched KEGG terms of up-regulated genes in KO neurons upon trehalose treatment

KEGG terms	*P-adj*	*P-value*	Genes
Steroid biosynthesis	7,05208E-13	3,54376E-15	SQLE,NSDHL,SC5D,DHCR24,MSMO1,DHCR7,HSD17B7,LSS,TM7SF2,FDFT1
Lysosome	1,67425E-07	1,68266E-09	ARSA,CD63,ASAH1,HEXB,CTSZ,HEXA,CLN3,CTSL,NPC2,GLB1,LAMP2,NEU1,MAN2B1
Other glycan degradation	7,7826E-05	1,17326E-06	GLB1,HEXB,HEXA,NEU1,MAN2B1
Terpenoid backbone biosynthesis	0,000173078	3,47896E-06	IDI1,MVK,PMVK,MVD,ACAT2
Sphingolipid metabolism	0,000620509	1,55907E-05	SMPD2,ARSA,ASAH1,SGPL1,GLB1,NEU1
Glutathione metabolism	0,00124717	3,76031E-05	MGST3,GSS,IDH1,MGST1,LAP3,PRDX6
PPAR signaling pathway	0,004672832	0,000164371	FADS2,ACADL,FABP7,DBI,PLIN2,ACSL3
Cysteine and methionine metabolism	0,005421161	0,000217936	TST,CBS,GSS,PHGDH,CDO1
Sphingolipid signaling pathway	0,00773801	0,00034996	GNA13,SMPD2,SGPL1,ASAH1,S1PR1,BID,TNFRSF1A
Fluid shear stress and atherosclerosis	0,017098413	0,000886299	CTSL,MGST3,MGST1,HMOX1,PLAT,NFE2L2,TNFRSF1A

Appendix Table S3. Significantly enriched GO terms of genes upregulated in WT and KO neurons upon trehalose treatment.

Cluster	ID	Term description	*P-adj*	*P-value*	Genes
WT_UP	GO:0061351	neural precursor cell proliferation	4,77E-05	1,83E-08	Kif1a,Optn,Zfp335,Wnt7a,Tafa1,Fgfr2,Rora,Id4,Plxnb2,Ctnna1,Slc16a2
WT_UP	GO:0007405	neuroblast proliferation	0,004398	3,37E-06	Zfp335,Tafa1,Fgfr2,Id4,Plxnb2,Ctnna1
WT_UP	GO:0044262	cellular carbohydrate metabolic process	0,024212	6,13E-05	Galk2,Pygb,Hk2,Rora,Ugp2,Stat3,Galk1,Gaa,Synj2
WT_UP	GO:0006575	cellular modified amino acid metabolic process	0,024212	6,59E-05	Crat,Gstm1,Slco1c1,Ctns,Dio2,Oplah,Slc16a2
WT_UP	GO:1904925	positive regulation of autophagy of mitochondrion in response to mitochondrial depolarization	0,024212	6,71E-05	Optn,Gba,Hk2
KO_UP	GO:0006643	membrane lipid metabolic process	5,59E-07	2,59E-10	Kdsr,Cers4,Tecr,St3gal4,Hexa,Elovl5,Smpd2,Smpdl3a,Gm2a,Hexb,Gpaa1,Neu1
KO_UP	GO:0006665	sphingolipid metabolic process	4,05E-06	3,74E-09	Kdsr,Cers4,Tecr,Hexa,Elovl5,Smpd2,Smpdl3a,Gm2a,Hexb,Neu1
KO_UP	GO:0030149	sphingolipid catabolic process	1,04E-05	1,44E-08	Hexa,Smpd2,Smpdl3a,Gm2a,Hexb,Neu1
KO_UP	GO:0046466	membrane lipid catabolic process	1,69E-05	3,12E-08	Hexa,Smpd2,Smpdl3a,Gm2a,Hexb,Neu1
KO_UP	GO:0046479	glycosphingolipid catabolic process	0,000335	7,75E-07	Hexa,Gm2a,Hexb,Neu1

Appendix Table S4. List of DEGs KO vs WT (from downregulated to upregulated genes)

Gene name	Gene description	log2FoldChange	*P-value*	*P-adj*	Diff. expression
Adm	Adrenomedullin	-0,75	0,000154123	0,051047739	DOWN
Haus7	HAUS augmin like complex subunit 7	-0,75	5,94154E-09	0,001	DOWN
Mecp2	Methyl-CpG binding protein 2	-0,75	0	0,001	DOWN
Eno1b	Enolase 1B	-0,443282634	5,69298E-05	0,030695164	DOWN
Eno1	Enolase 1	-0,39193667	0,000234269	0,06661656	DOWN
Ldha	Lactate dehydrogenase A	-0,352662394	0,000148045	0,051047739	DOWN
Poll	DNA polymerase lambda	-0,332736299	0,000108246	0,041374192	DOWN
Rfc2	Replication factor c subunit 2	-0,324714096	6,48235E-06	0,008026497	DOWN
Mif	Macrophage migration inhibitory factor	-0,318728531	1,80229E-05	0,011938915	DOWN
Nsdhl	NAD(P) dependent steroid dehydrogenase-like	-0,308591452	7,07599E-06	0,008026497	DOWN
Gapdh	Glyceraldehyde-3-phosphate dehydrogenase	-0,235004344	0,000247244	0,067439502	DOWN
Prelid1	PRELI domain containing 1	-0,208320215	0,000167896	0,051902245	DOWN
Phc2	Polyhomeotic homolog 2	-0,192300756	5,95766E-05	0,030695164	DOWN
Cdv3	Carnitine deficiency-associated gene expressed In ventricle 3	0,158435947	8,65484E-06	0,008026497	UP
Cep120	Centrosomal protein 120	0,208294171	1,28771E-05	0,010239336	UP
Ivns1abp	Influenza virus NS1A binding protein	0,213913606	0,000167481	0,051902245	UP
Retreg1	Reticulophagy regulator 1	0,228603493	4,5686E-06	0,007061535	UP
Foxp1	Forkhead box P1	0,258324896	0,000111533	0,041374192	UP
Usp6nl	USP6 N-terminal like	0,261365434	9,82375E-05	0,041374192	UP
Nab1	NGFI-A binding protein 1	0,269556192	9,22351E-05	0,04073277	UP
B3galt1	Beta-1,3-galactosyltransferase 1	0,27564971	7,72981E-05	0,03584312	UP
Kdm1b	Lysine demethylase 1B	0,284137873	0,000223512	0,06661656	UP
Rnf150	Ring finger protein 150	0,29983626	0,000237044	0,06661656	UP
Kazn	Kazrin, periplakin interacting protein	0,300699296	1,74763E-05	0,011938915	UP
Zfp608	Zinc finger protein 608	0,32769365	0,000385678	0,096669562	UP
Nek7	NIMA related kinase 7	0,361734137	1,24063E-06	0,002301117	UP
Slc24a2	Solute carrier family 24 member 2	0,376459917	0,000108208	0,041374192	UP
Cntnap4	Contactin associated protein family member 4	0,381764957	0,000313429	0,083049808	UP
Lonrf1	LON peptidase N-terminal domain and ring finger 1	0,394141173	4,42783E-05	0,025664801	UP
Ank1	Ankyrin 1	0,402430051	7,12689E-05	0,034786746	UP
Lgi2	Leucine rich repeat LGI family member 2	0,455318194	1,48781E-08	0,001	UP
Zmat4	Zinc finger matrin-type 4	0,456564799	1,32491E-05	0,010239336	UP
Pde10a	Phosphodiesterase 10A	0,475453466	3,19287E-05	0,019740452	UP
Oprd1	Opioid receptor delta 1	0,482208156	0,000150931	0,051047739	UP
Scn4b	Sodium voltage-gated channel beta subunit 4	0,595172165	0,000328498	0,084624681	UP
Nr4a3	Nuclear receptor subfamily 4 group A member 3	0,716876663	7,95692E-06	0,008026497	UP
Irak1	Interleukin 1 receptor associated kinase 1	0,75	8,59457E-64	0,001	UP

Appendix Figure S1. Mecp2-deficient neurons do not display overt lysosomal defects. (A) Representative TEM micrographs of KO cortical neurons (14 DIV) incubated with 20 uM leupeptin for 24 h, showing the appearance of degradative organelles containing electron-dense material in the cell bodies and neurites. **(B)** Representative western blot from lysates of WT and KO cortical neurons (14 DIV). LAMP1 intensity was quantified by densitometric analysis and normalized on GAPDH intensity. Data are expressed as median ± min/max (n= 6 embryos from two independent experiments). Mann-Whitney test.

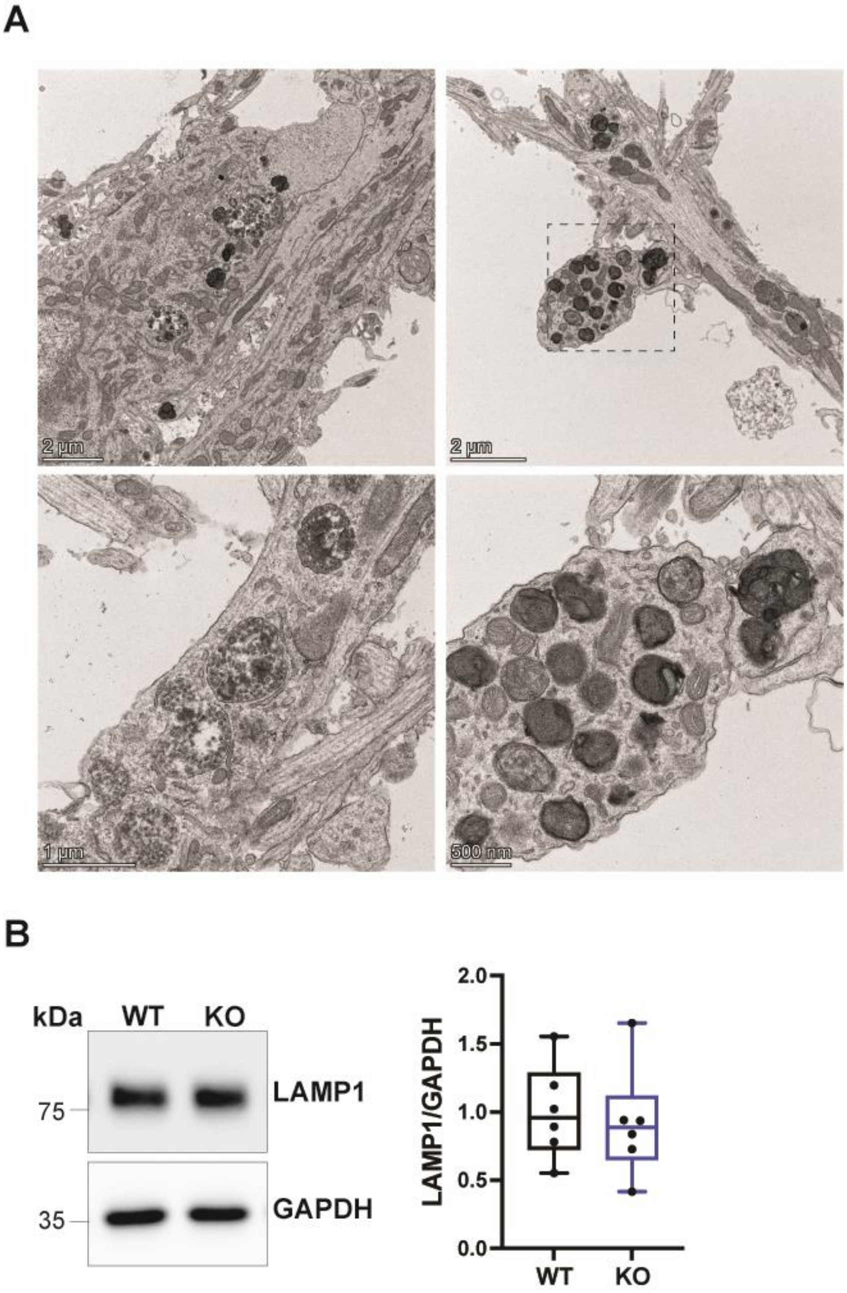

Appendix Figure S2. Ethanolamine treatment enhances LC3B-II lipidation. (A) Left panel. Representative western blot from lysates of WT cortical neurons (14 DIV) under resting condition (-) or supplemented for 16h with increasing concentration of ethanolamine in the cell medium. p62, LC3B-I and LC3B-II signals are shown. GAPDH was used as loading control. 10 mM ethanolamine was able to increase the levels of LC3B-II. Right panel. Representative western blot from lysates of WT and KO cortical neurons (14 DIV) under resting condition or incubated with 10 mM ethanolamine for 16h. p62, LC3B-I and LC3B-II signals are shown. GAPDH was used as loading control.

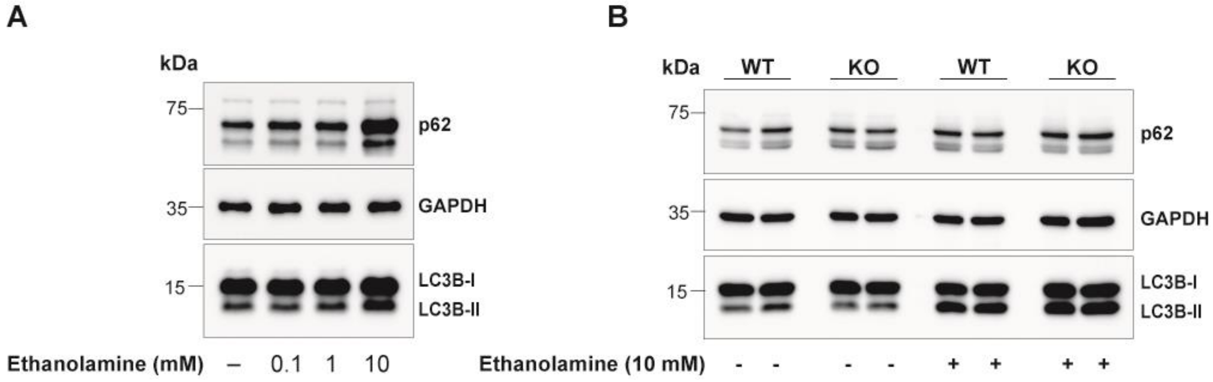

Appendix Figure S3. Autophagy induction by trehalose recovers neuronal complexity. **(A)** The average number of intersections per each radial distance from the soma of WT untreated (N=41 from 3 embryos) or treated (N=43 from 3 embryos) and KO untreated (N=56 from 3 embryos) or treated (N=45 from 3 embryos) neurons is reported. **(B)** Quantification of the presynaptic terminal area on TEM micrographs of WT and KO cortical neurons (14 DIV). Data are expressed as violin plots, median ± quartiles (n > 60 cells analyzed/condition for each experiment, from three independent experiments). **(C)** Left: representative traces of mEPSCs recordings from untreated and trehalose treated (48h) WT and KO cortical neurons (DIV 14). Right: quantification of mEPSCs features (amplitude, area, decay time, frequency). Data are expressed as mean ± SEM (20-27 neurons from 6 embryos/genotype, four independent experiments). Two-way ANOVA with Holm-Šídák's multiple comparisons test (*p<0.05, **p<0.01).

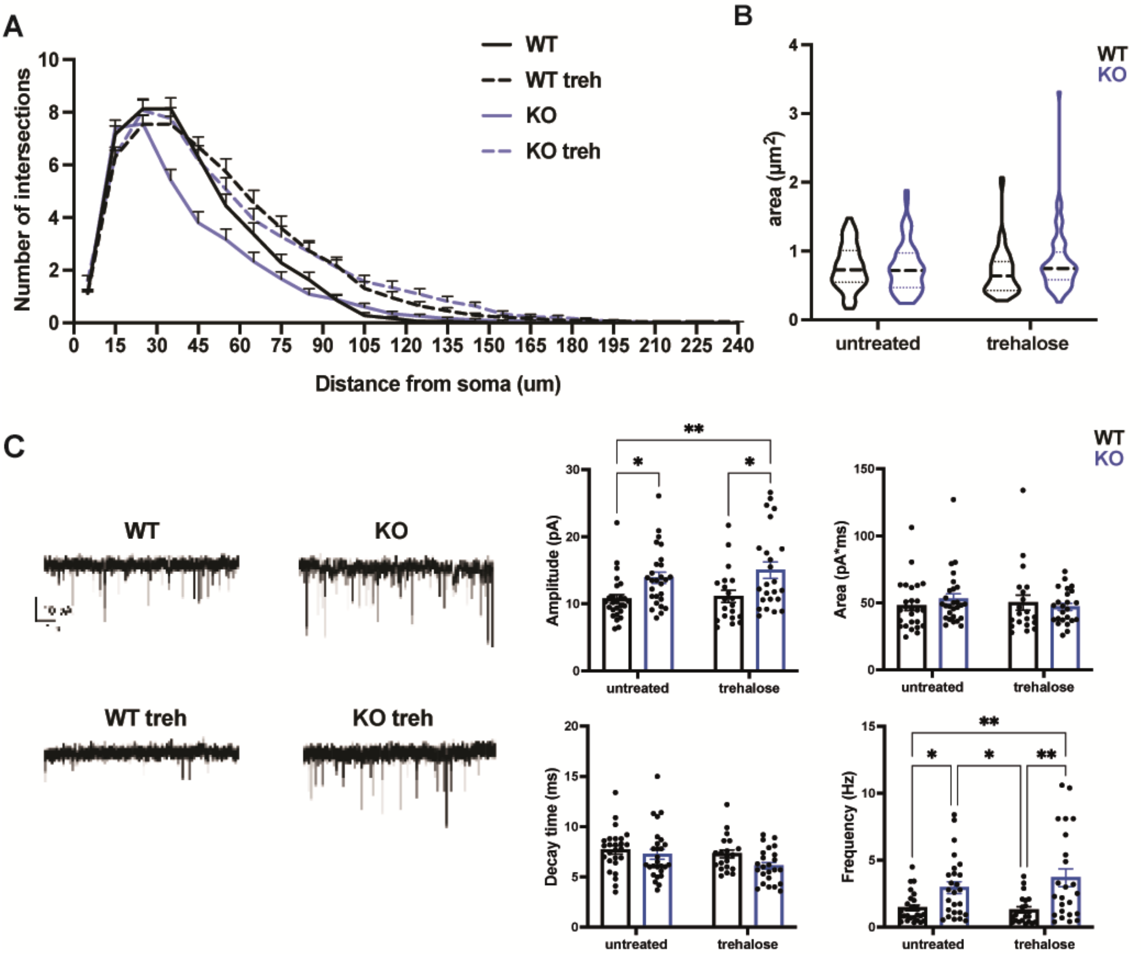

Supplement: Supplementary file 1 — Appendix [file 44321_2024_151_MOESM1_ESM.pdf]
