# Supplementary figures and images for: Unraveling autophagic imbalances and therapeutic insights in Mecp2-deficient models
Source: EMBO Mol Med. 2024 Oct 14;16(11):2795–826. doi: 10.1038/s44321-024-00151-w (PMC11555085; doi:10.1038/s44321-024-00151-w)

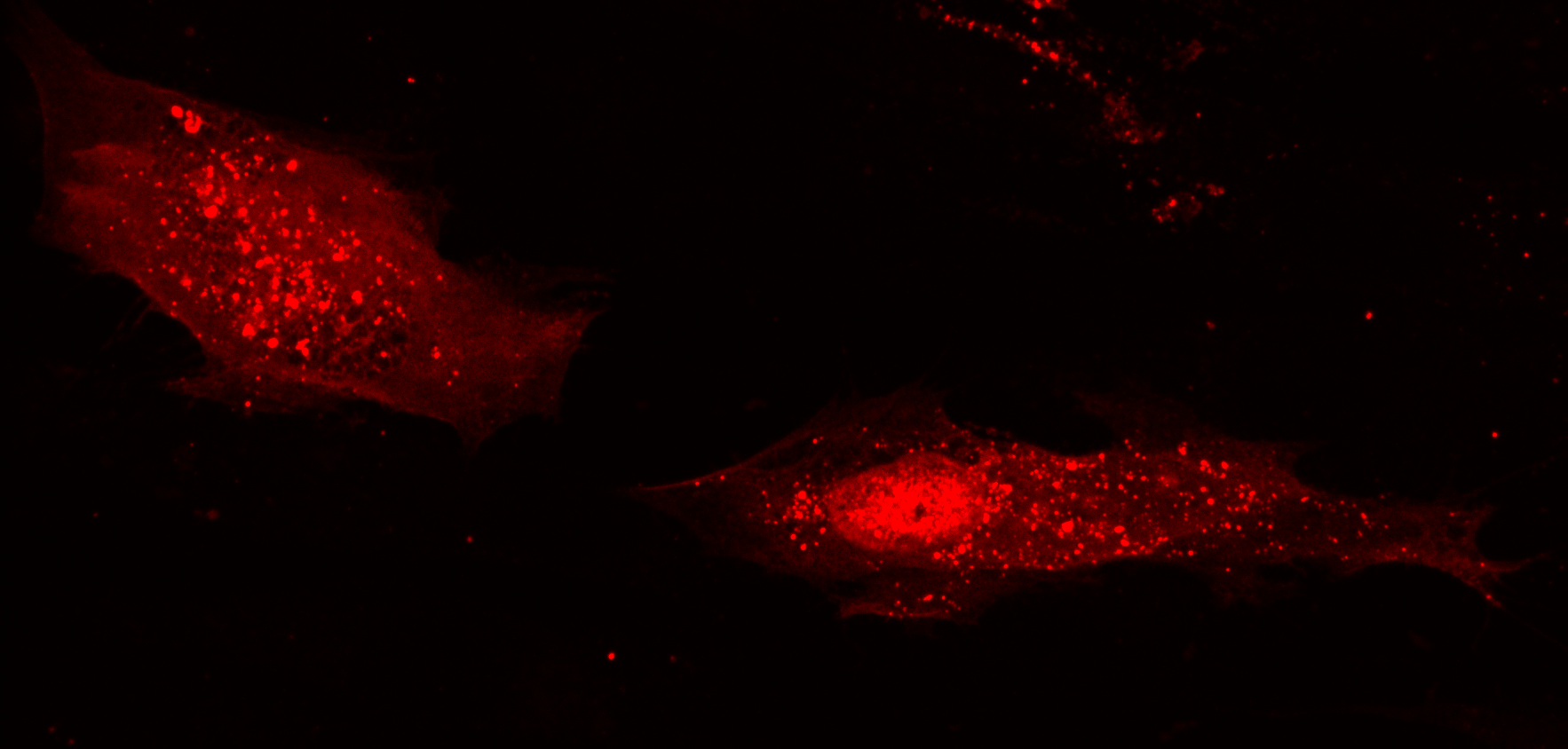

Supplement: Supplementary file 3 — Source data Fig. 1 [file 44321_2024_151_MOESM3_ESM.zip › EMM-2023-19183_SourceDataForFigure 1/1F/TRITC-EGFP-mCherry-LC3B.tif]

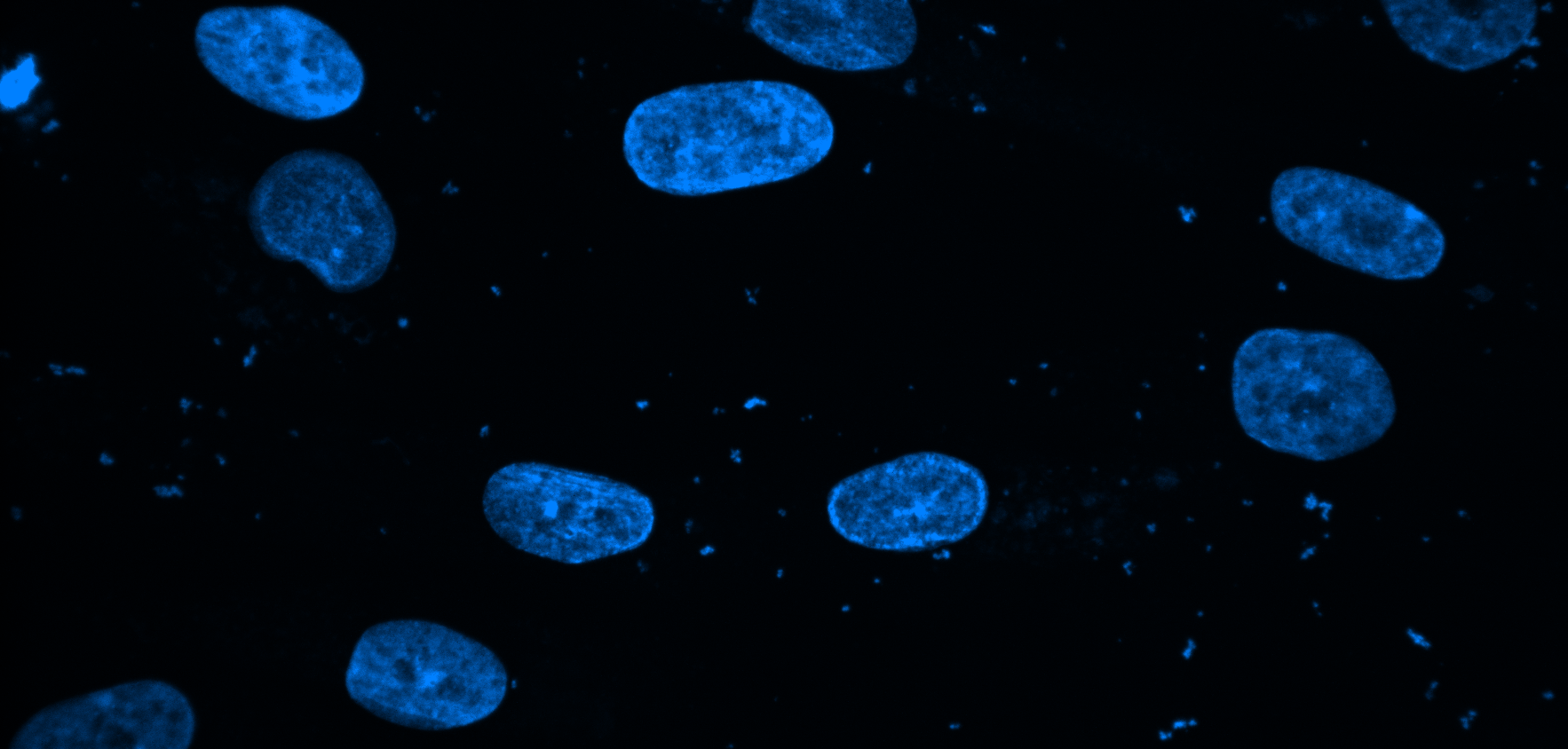

Supplement: Supplementary file 3 — Source data Fig. 1 [file 44321_2024_151_MOESM3_ESM.zip › EMM-2023-19183_SourceDataForFigure 1/1F/DAPI.tif]

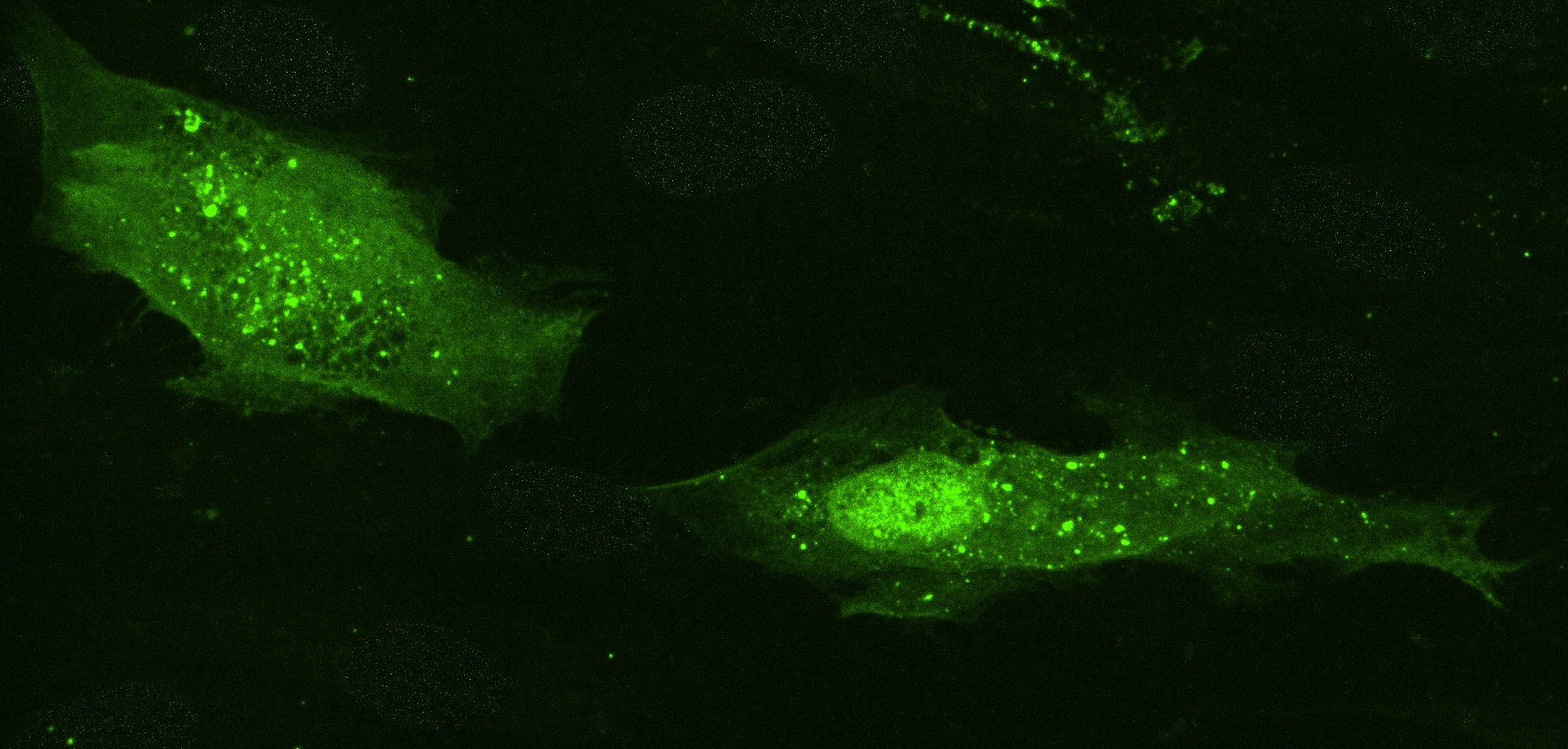

Supplement: Supplementary file 3 — Source data Fig. 1 [file 44321_2024_151_MOESM3_ESM.zip › EMM-2023-19183_SourceDataForFigure 1/1F/FITC-EGFP-mCherry-LC3B.tif]

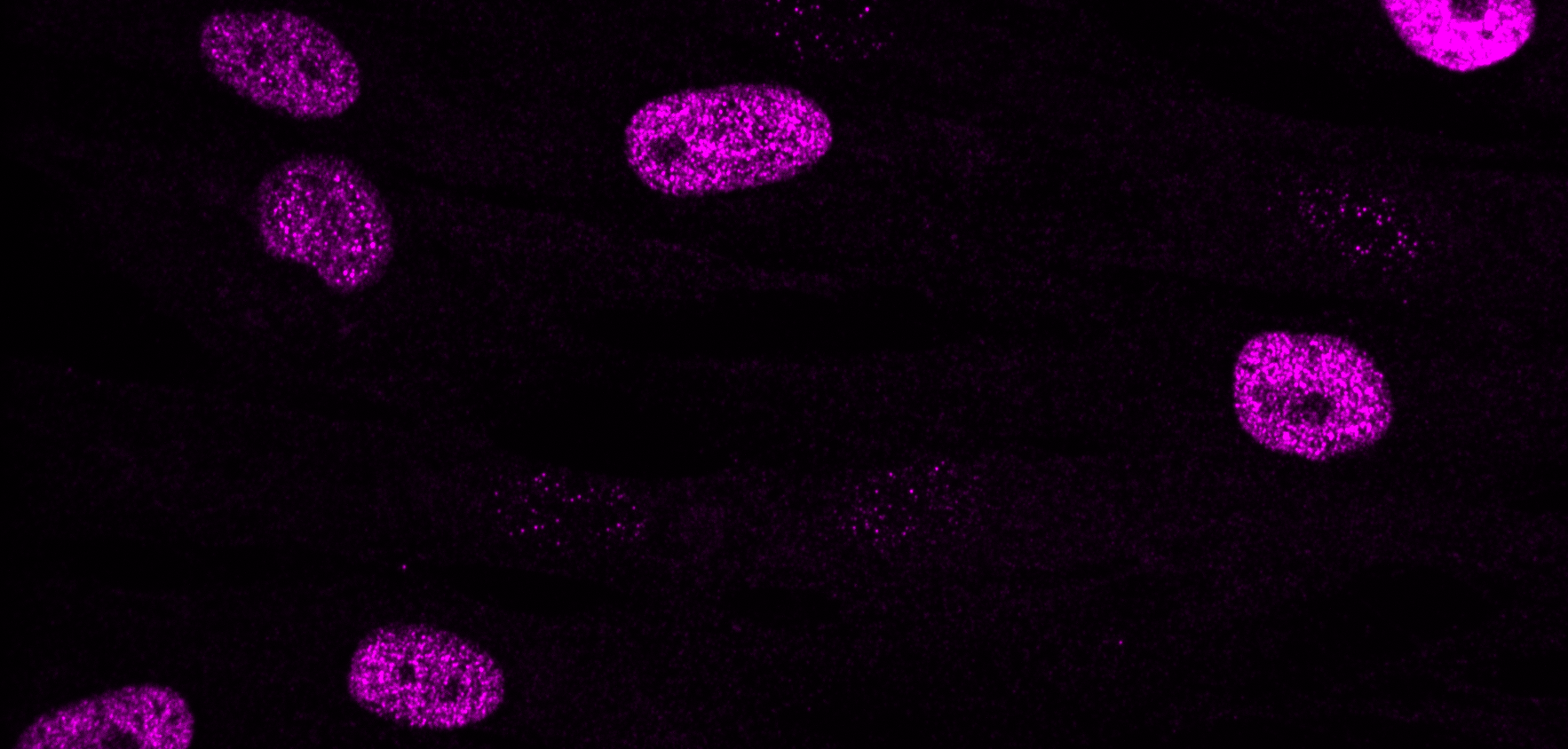

Supplement: Supplementary file 3 — Source data Fig. 1 [file 44321_2024_151_MOESM3_ESM.zip › EMM-2023-19183_SourceDataForFigure 1/1F/MeCP2.tif]

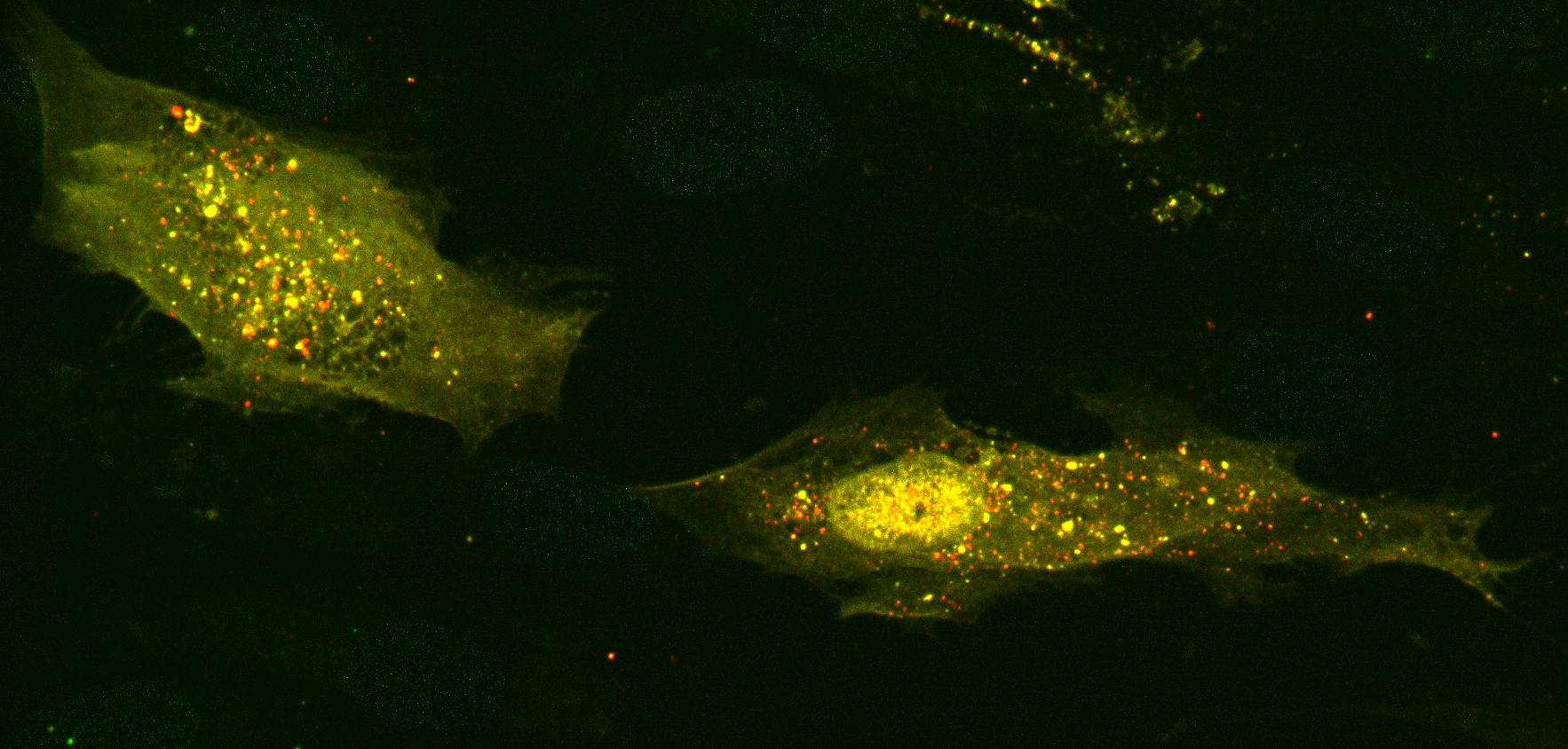

Supplement: Supplementary file 3 — Source data Fig. 1 [file 44321_2024_151_MOESM3_ESM.zip › EMM-2023-19183_SourceDataForFigure 1/1F/MERGE.tif]

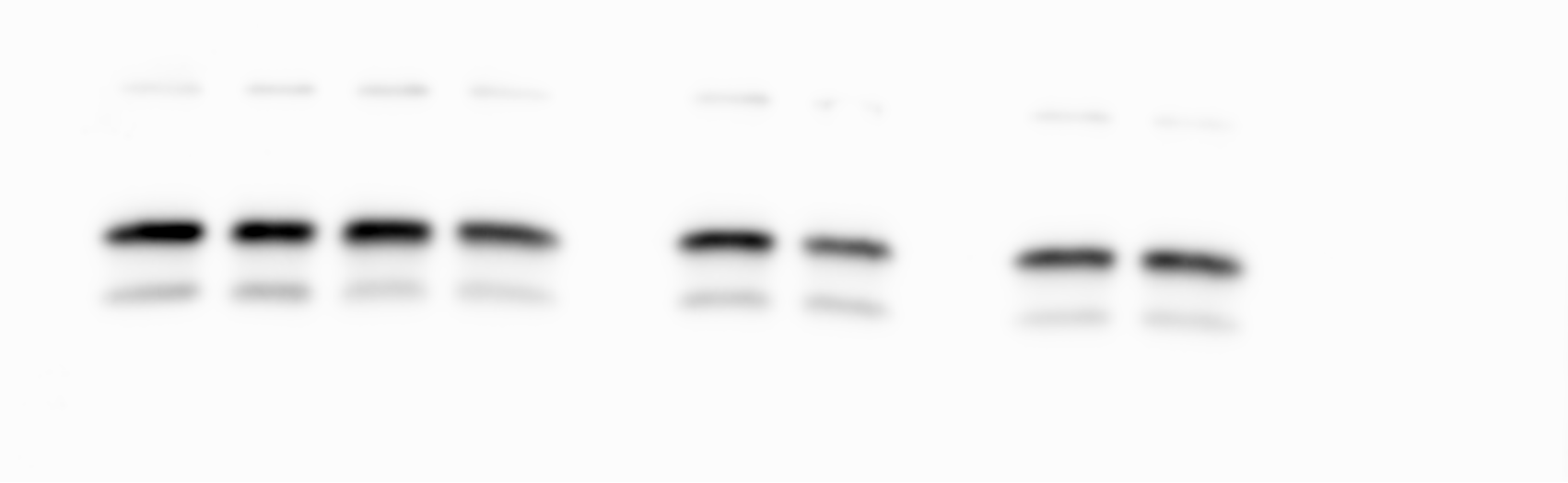

Supplement: Supplementary file 3 — Source data Fig. 1 [file 44321_2024_151_MOESM3_ESM.zip › EMM-2023-19183_SourceDataForFigure 1/1A/LC3-I uncropped.tif]

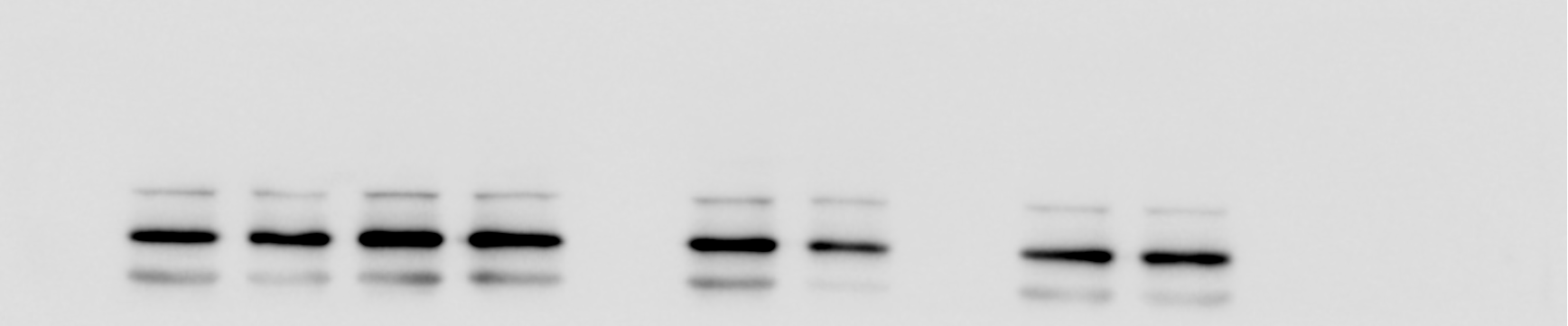

Supplement: Supplementary file 3 — Source data Fig. 1 [file 44321_2024_151_MOESM3_ESM.zip › EMM-2023-19183_SourceDataForFigure 1/1A/p62 uncropped.tif]

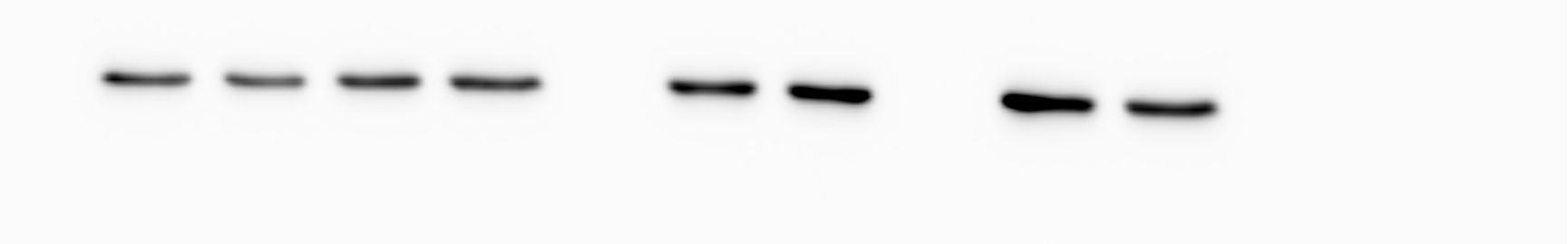

Supplement: Supplementary file 3 — Source data Fig. 1 [file 44321_2024_151_MOESM3_ESM.zip › EMM-2023-19183_SourceDataForFigure 1/1A/GAPDH uncropped.tif]

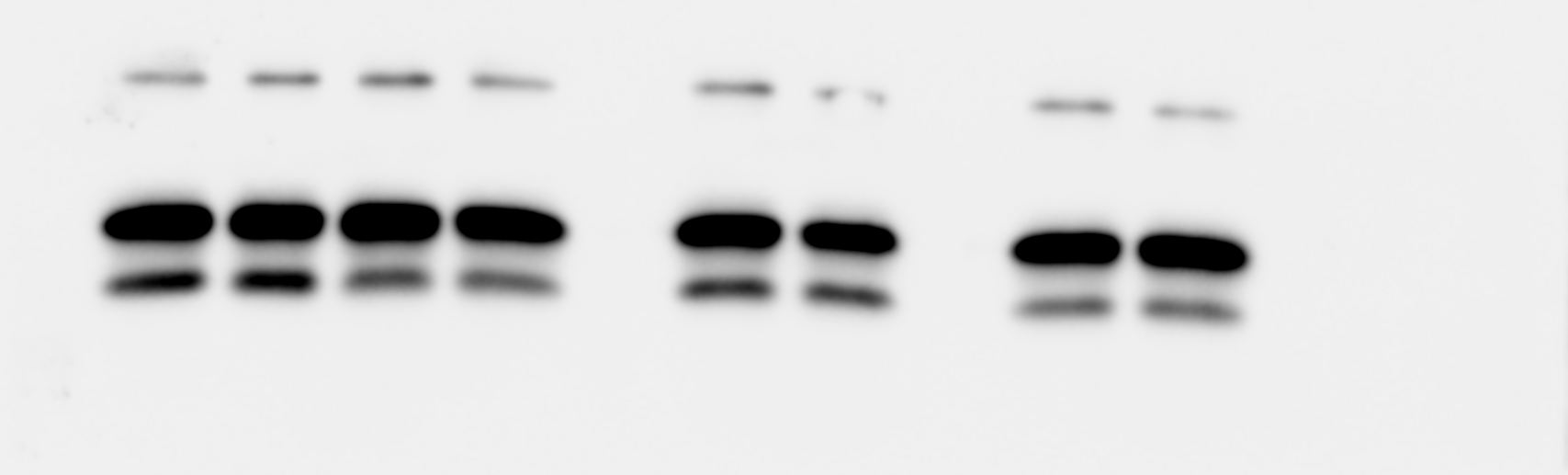

Supplement: Supplementary file 3 — Source data Fig. 1 [file 44321_2024_151_MOESM3_ESM.zip › EMM-2023-19183_SourceDataForFigure 1/1A/LC3-II uncropped.tif]

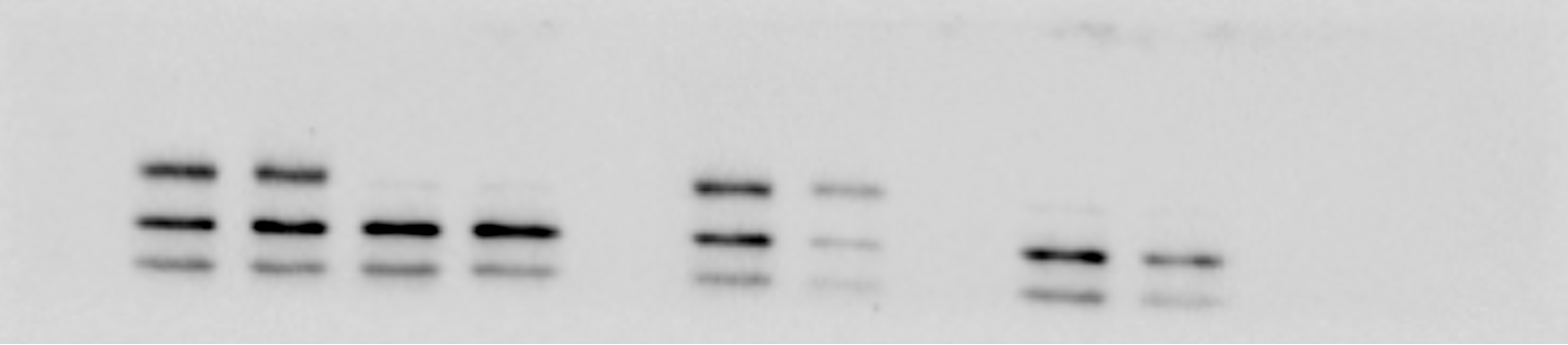

Supplement: Supplementary file 3 — Source data Fig. 1 [file 44321_2024_151_MOESM3_ESM.zip › EMM-2023-19183_SourceDataForFigure 1/1A/MeCP2 uncropped.tif]

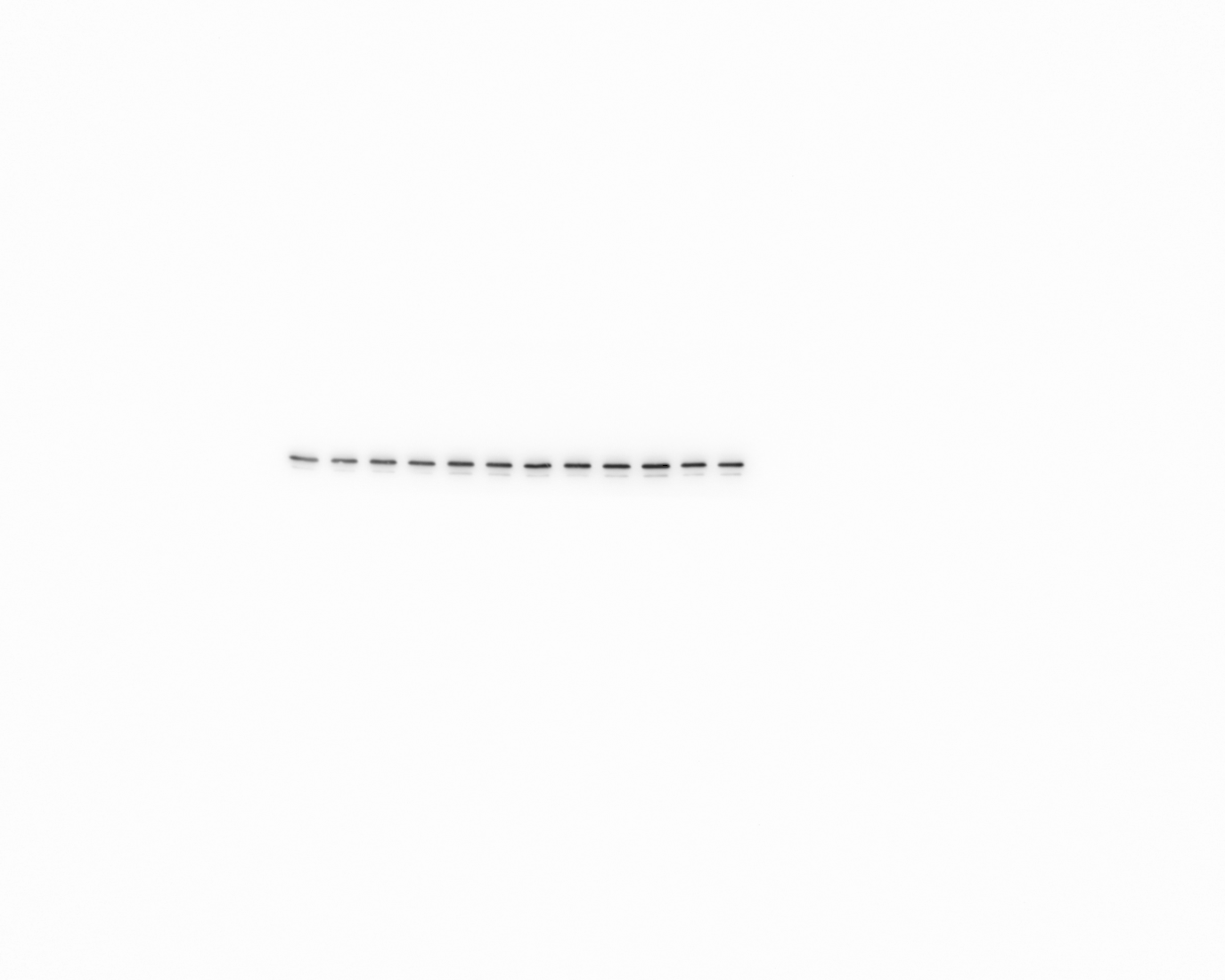

Supplement: Supplementary file 3 — Source data Fig. 1 [file 44321_2024_151_MOESM3_ESM.zip › EMM-2023-19183_SourceDataForFigure 1/1B/GAPDH.tif]

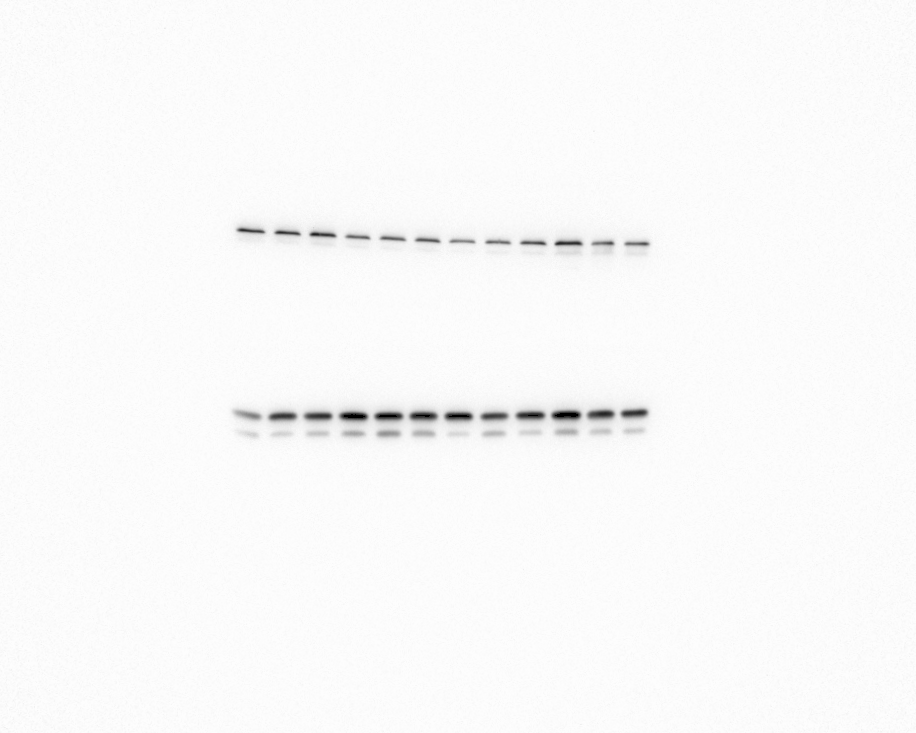

Supplement: Supplementary file 3 — Source data Fig. 1 [file 44321_2024_151_MOESM3_ESM.zip › EMM-2023-19183_SourceDataForFigure 1/1B/P62 - LC3B.tif]

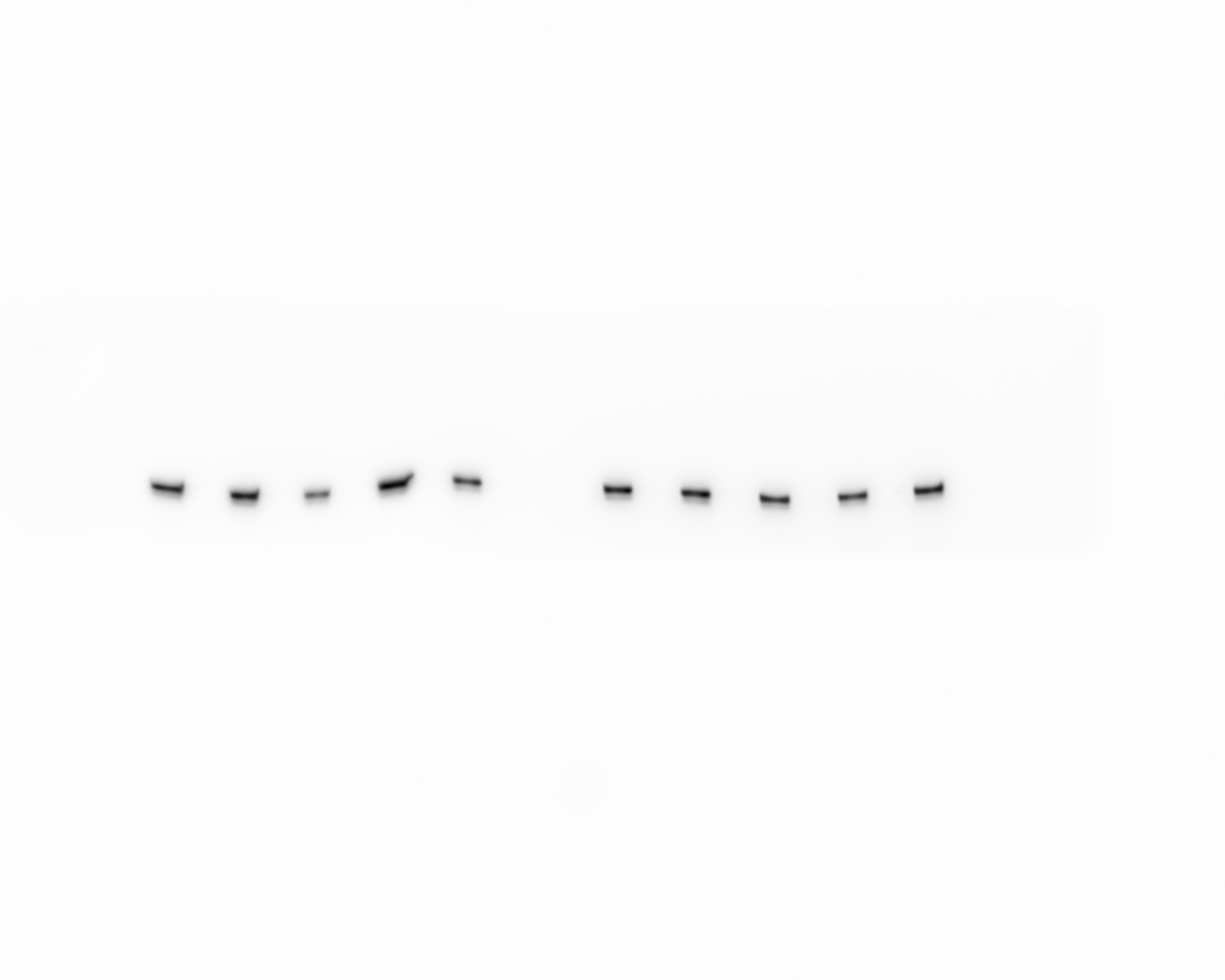

Supplement: Supplementary file 3 — Source data Fig. 1 [file 44321_2024_151_MOESM3_ESM.zip › EMM-2023-19183_SourceDataForFigure 1/1B/MeCP2.tif]

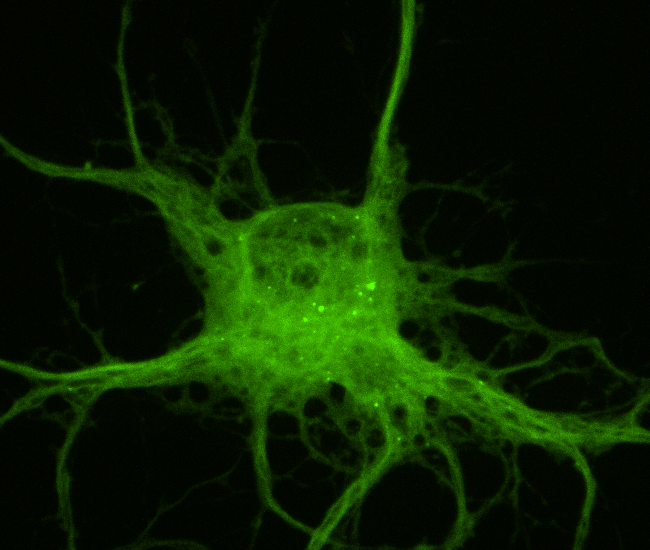

Supplement: Supplementary file 3 — Source data Fig. 1 [file 44321_2024_151_MOESM3_ESM.zip › EMM-2023-19183_SourceDataForFigure 1/1E/FITC-EGFP-mCherry-LC3B_KO13.tif]

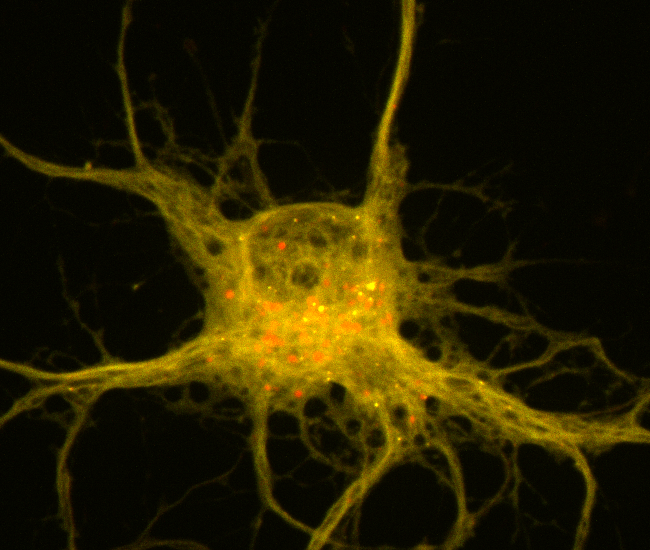

Supplement: Supplementary file 3 — Source data Fig. 1 [file 44321_2024_151_MOESM3_ESM.zip › EMM-2023-19183_SourceDataForFigure 1/1E/MERGE-EGFP-mCherry-LC3B_ KO4.tif]

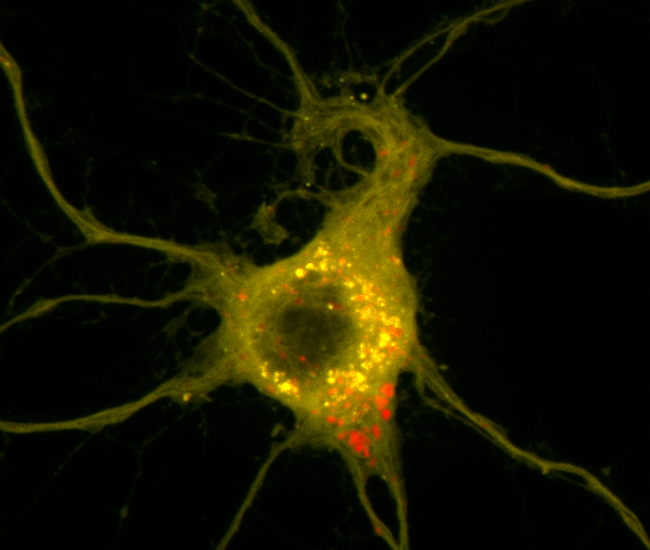

Supplement: Supplementary file 3 — Source data Fig. 1 [file 44321_2024_151_MOESM3_ESM.zip › EMM-2023-19183_SourceDataForFigure 1/1E/MERGE-EGFP-mCherry-LC3B_WT7.tif]

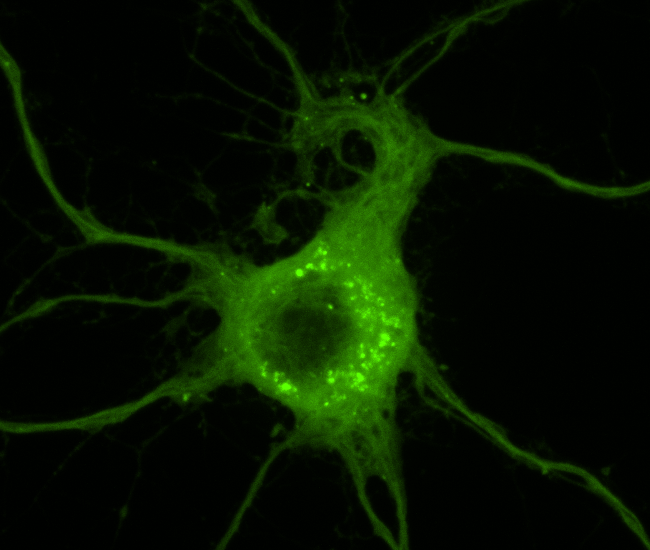

Supplement: Supplementary file 3 — Source data Fig. 1 [file 44321_2024_151_MOESM3_ESM.zip › EMM-2023-19183_SourceDataForFigure 1/1E/FITC-EGFP-mCherry-LC3B_WT7.tif]

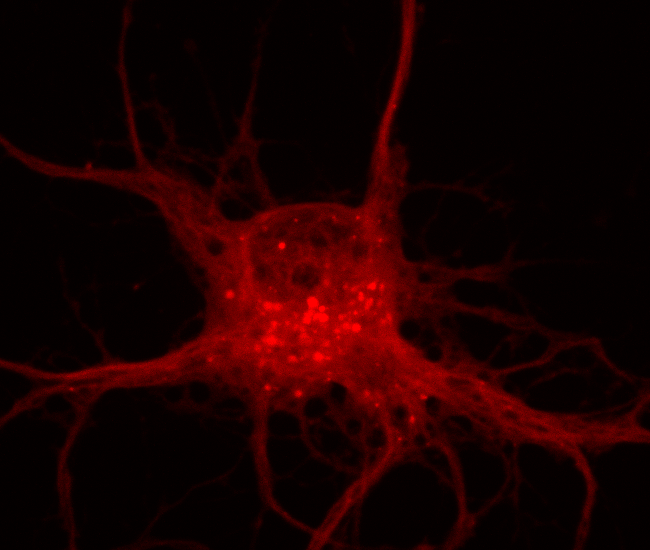

Supplement: Supplementary file 3 — Source data Fig. 1 [file 44321_2024_151_MOESM3_ESM.zip › EMM-2023-19183_SourceDataForFigure 1/1E/TRITC-EGFP-mCherry-LC3B_KO13.tif]

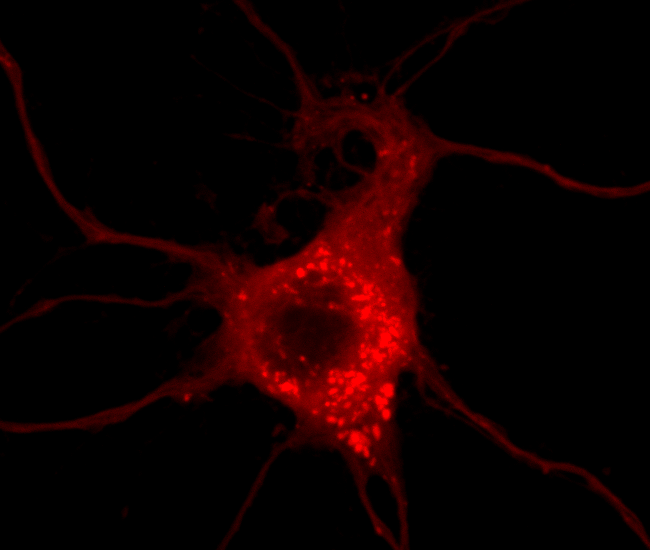

Supplement: Supplementary file 3 — Source data Fig. 1 [file 44321_2024_151_MOESM3_ESM.zip › EMM-2023-19183_SourceDataForFigure 1/1E/TRITC-EGFP-mCherry-LC3B_WT7.tif]

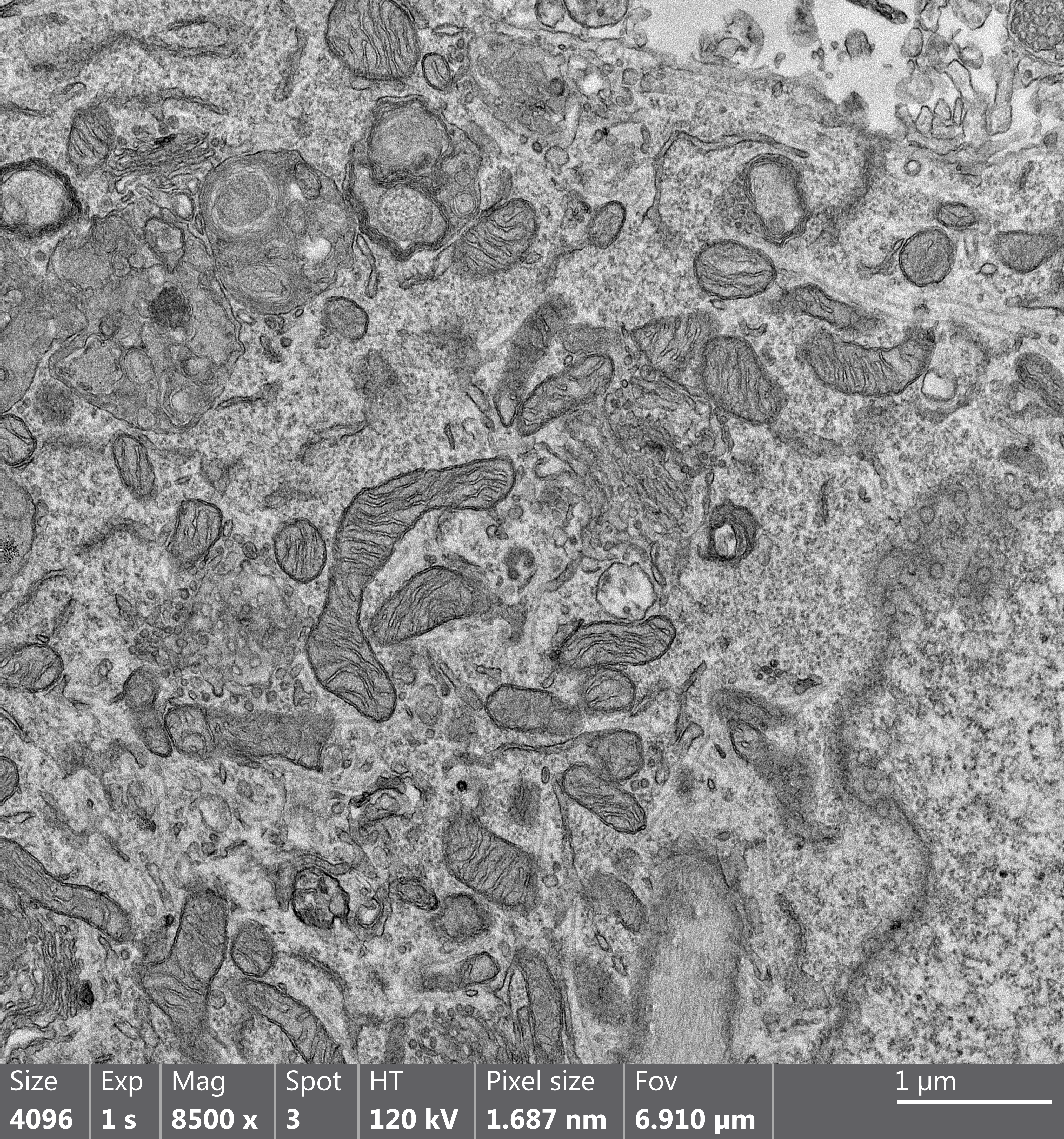

Supplement: Supplementary file 3 — Source data Fig. 1 [file 44321_2024_151_MOESM3_ESM.zip › EMM-2023-19183_SourceDataForFigure 1/1D/N_WT 0003.tif]

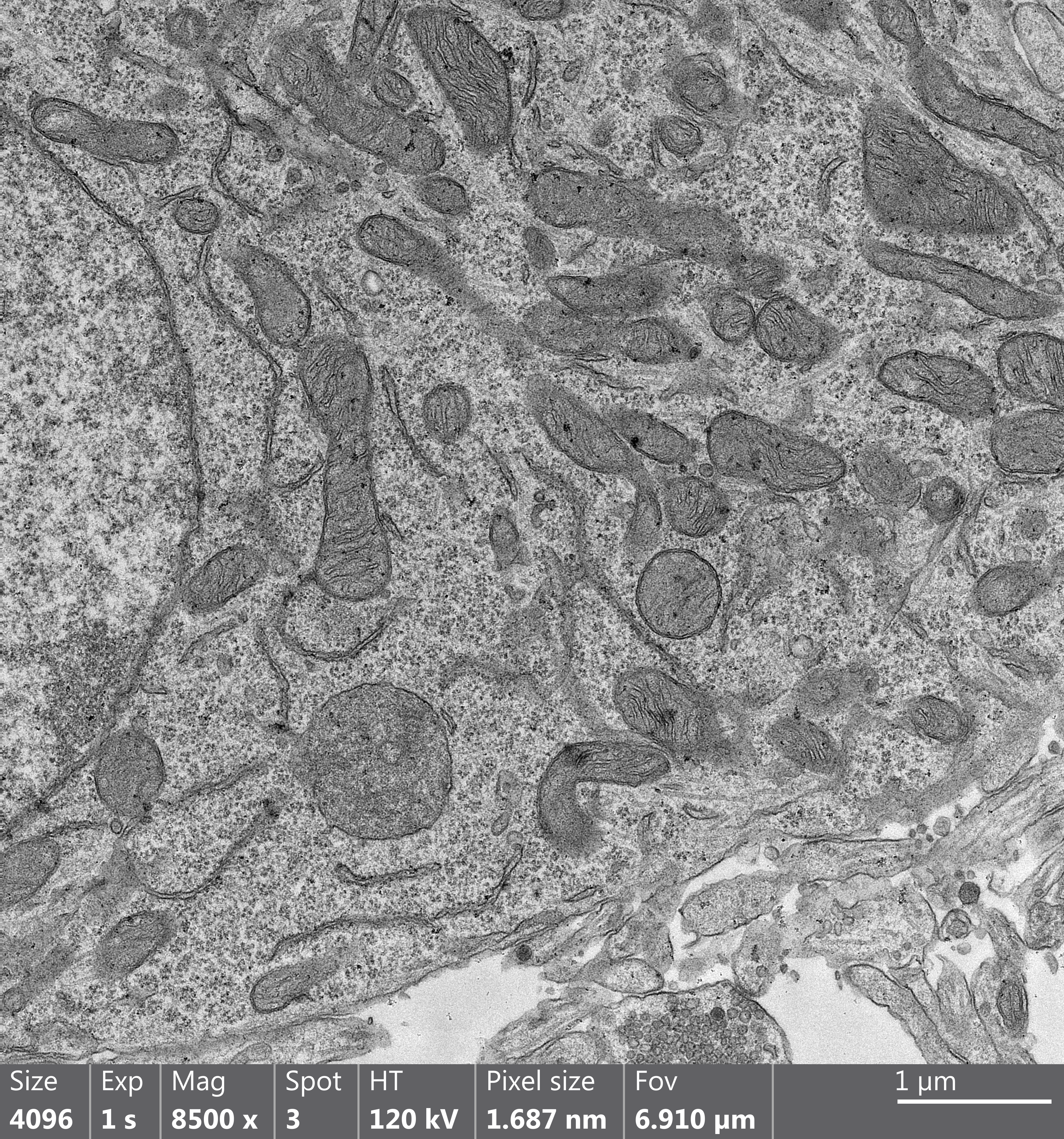

Supplement: Supplementary file 3 — Source data Fig. 1 [file 44321_2024_151_MOESM3_ESM.zip › EMM-2023-19183_SourceDataForFigure 1/1D/N_KO 0010.tif]

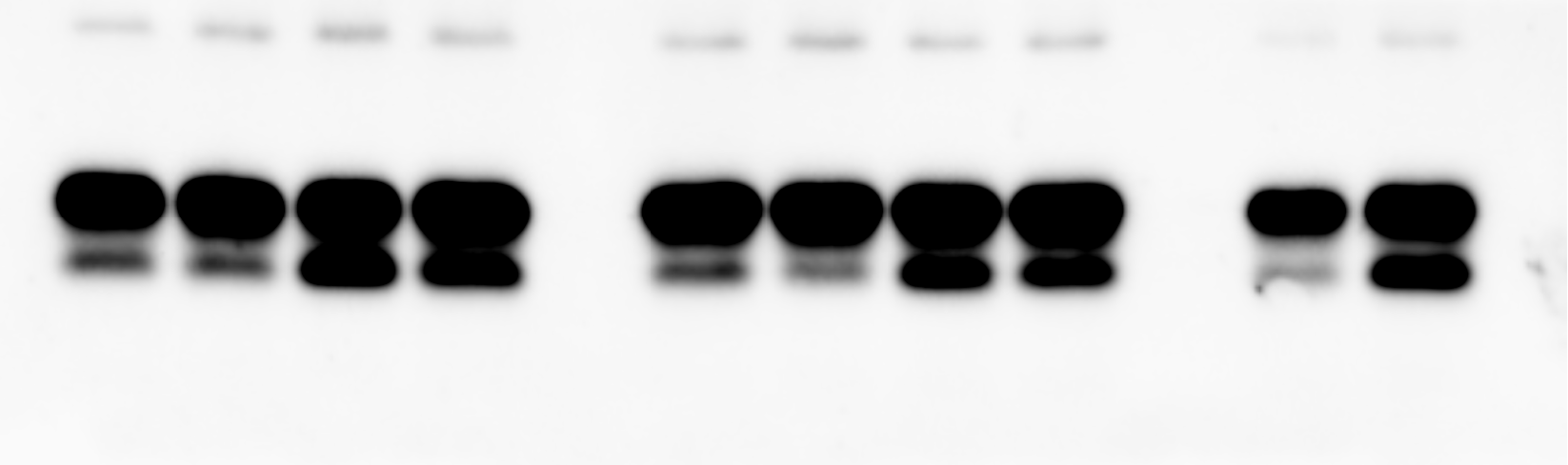

Supplement: Supplementary file 3 — Source data Fig. 1 [file 44321_2024_151_MOESM3_ESM.zip › EMM-2023-19183_SourceDataForFigure 1/1C/LC3 uncropped.tif]

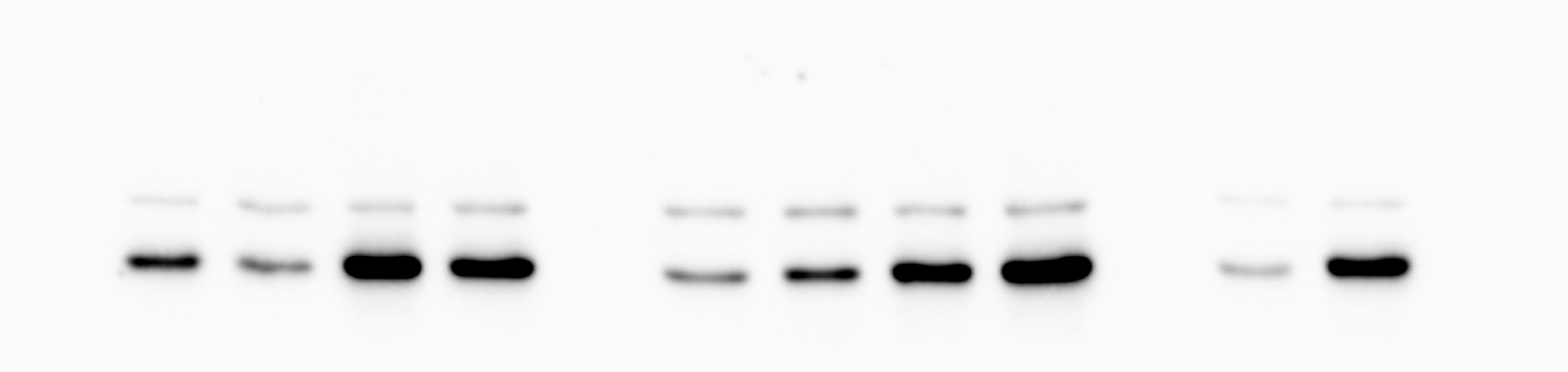

Supplement: Supplementary file 3 — Source data Fig. 1 [file 44321_2024_151_MOESM3_ESM.zip › EMM-2023-19183_SourceDataForFigure 1/1C/p62 uncropped.tif]

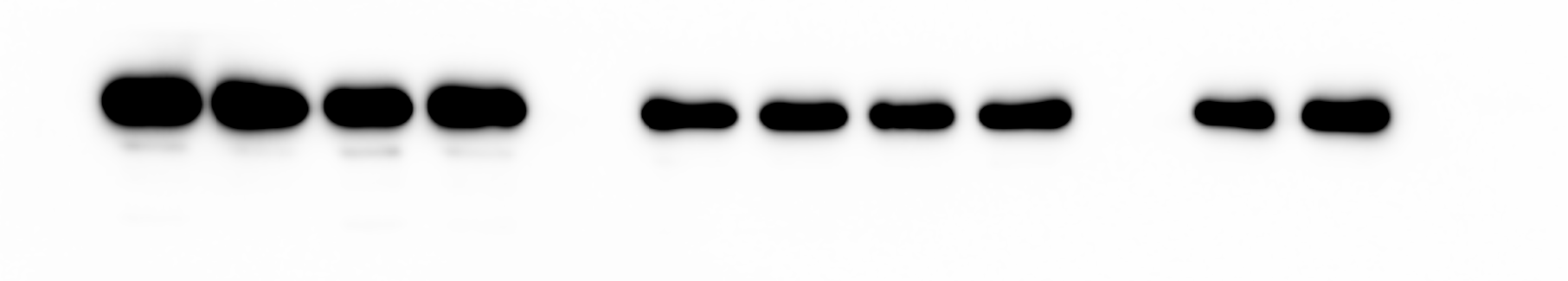

Supplement: Supplementary file 3 — Source data Fig. 1 [file 44321_2024_151_MOESM3_ESM.zip › EMM-2023-19183_SourceDataForFigure 1/1C/GAPDH uncropped lower.tif]

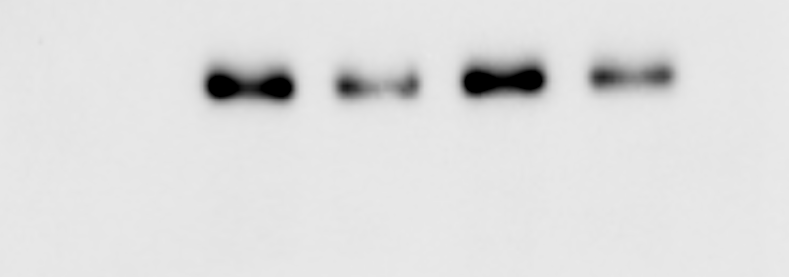

Supplement: Supplementary file 3 — Source data Fig. 1 [file 44321_2024_151_MOESM3_ESM.zip › EMM-2023-19183_SourceDataForFigure 1/1C/MeCP2 uncropped.tif]

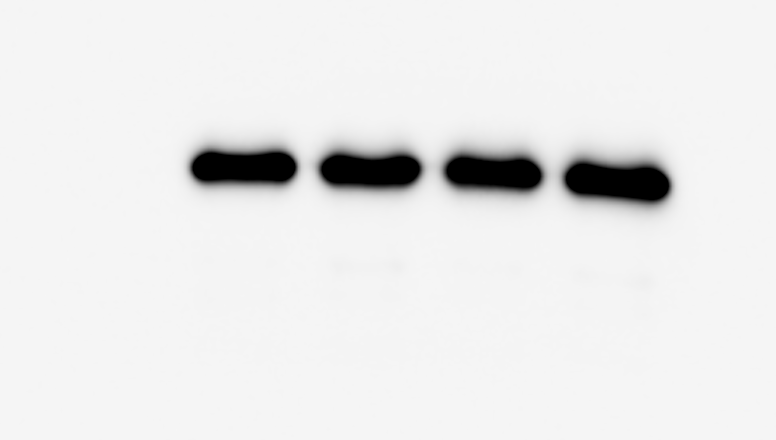

Supplement: Supplementary file 3 — Source data Fig. 1 [file 44321_2024_151_MOESM3_ESM.zip › EMM-2023-19183_SourceDataForFigure 1/1C/GAPDH uncropped upper.tif]

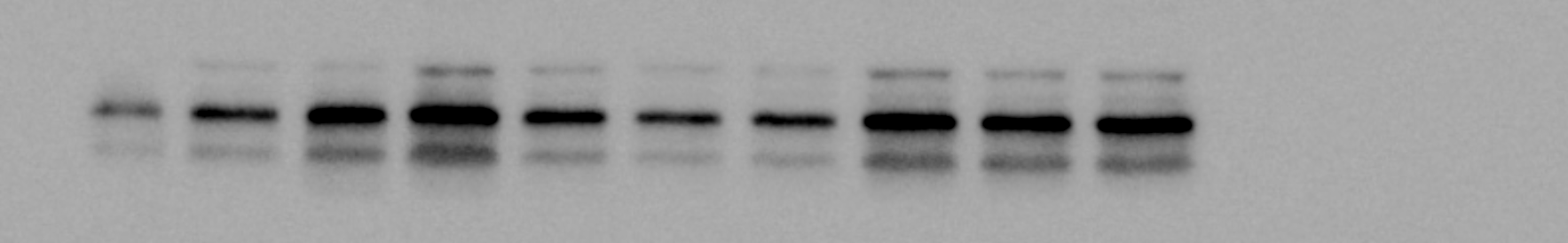

Supplement: Supplementary file 4 — Source data Fig. 2 [file 44321_2024_151_MOESM4_ESM.zip › EMM-2023-19183_SourceDataForFigure 2 /2A/p62.tif]

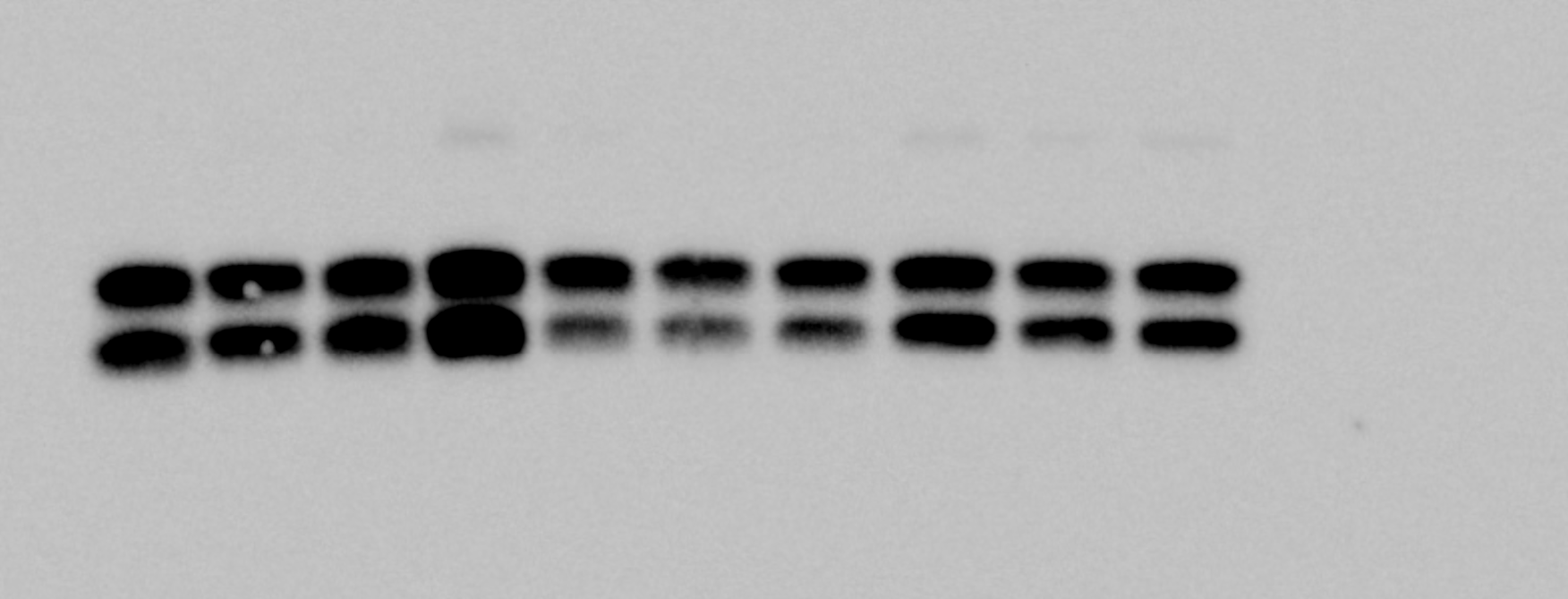

Supplement: Supplementary file 4 — Source data Fig. 2 [file 44321_2024_151_MOESM4_ESM.zip › EMM-2023-19183_SourceDataForFigure 2 /2A/LC3.tif]

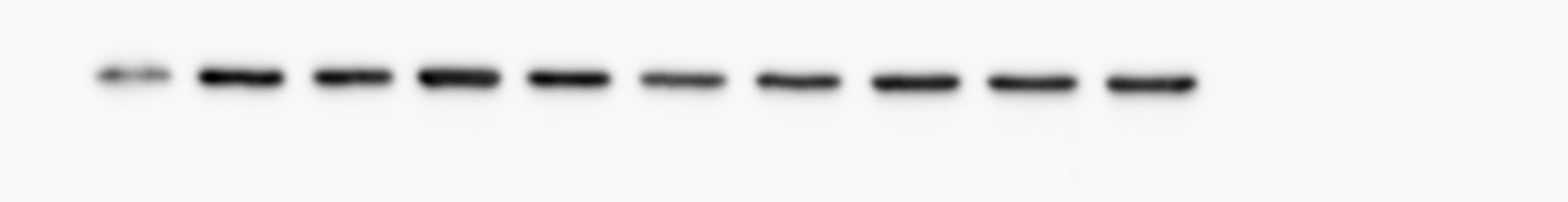

Supplement: Supplementary file 4 — Source data Fig. 2 [file 44321_2024_151_MOESM4_ESM.zip › EMM-2023-19183_SourceDataForFigure 2 /2A/GAPDH.tif]

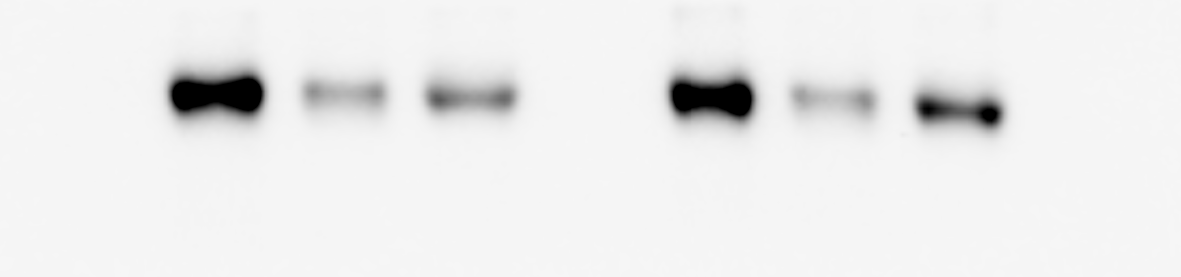

Supplement: Supplementary file 4 — Source data Fig. 2 [file 44321_2024_151_MOESM4_ESM.zip › EMM-2023-19183_SourceDataForFigure 2 /2C/p-p70S6K1.tif]

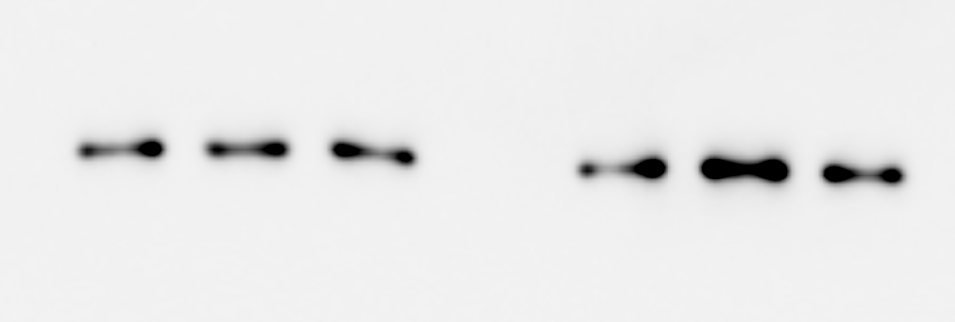

Supplement: Supplementary file 4 — Source data Fig. 2 [file 44321_2024_151_MOESM4_ESM.zip › EMM-2023-19183_SourceDataForFigure 2 /2C/GAPDH upper.tif]

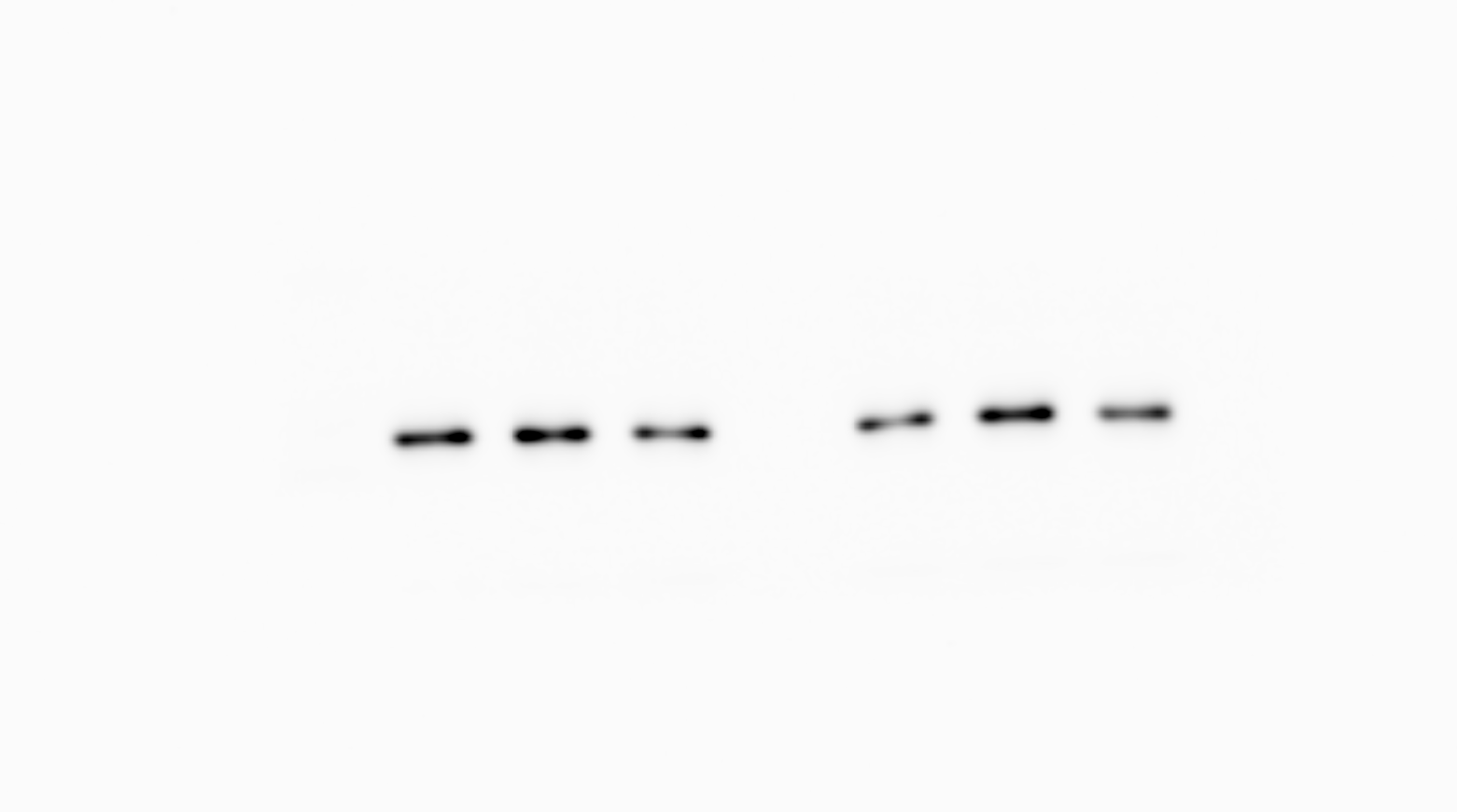

Supplement: Supplementary file 4 — Source data Fig. 2 [file 44321_2024_151_MOESM4_ESM.zip › EMM-2023-19183_SourceDataForFigure 2 /2C/GAPDH lower.tif]

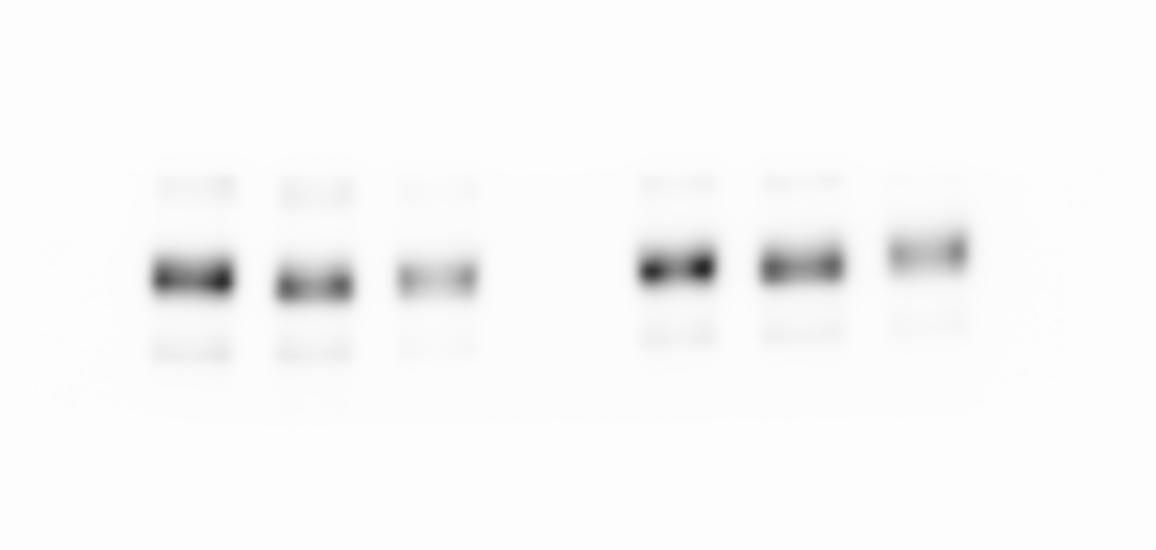

Supplement: Supplementary file 4 — Source data Fig. 2 [file 44321_2024_151_MOESM4_ESM.zip › EMM-2023-19183_SourceDataForFigure 2 /2C/p70S6K1.tif]

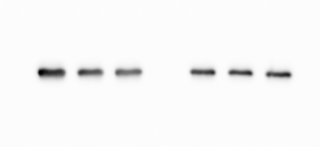

Supplement: Supplementary file 4 — Source data Fig. 2 [file 44321_2024_151_MOESM4_ESM.zip › EMM-2023-19183_SourceDataForFigure 2 /2B/Gapdh.tiff]

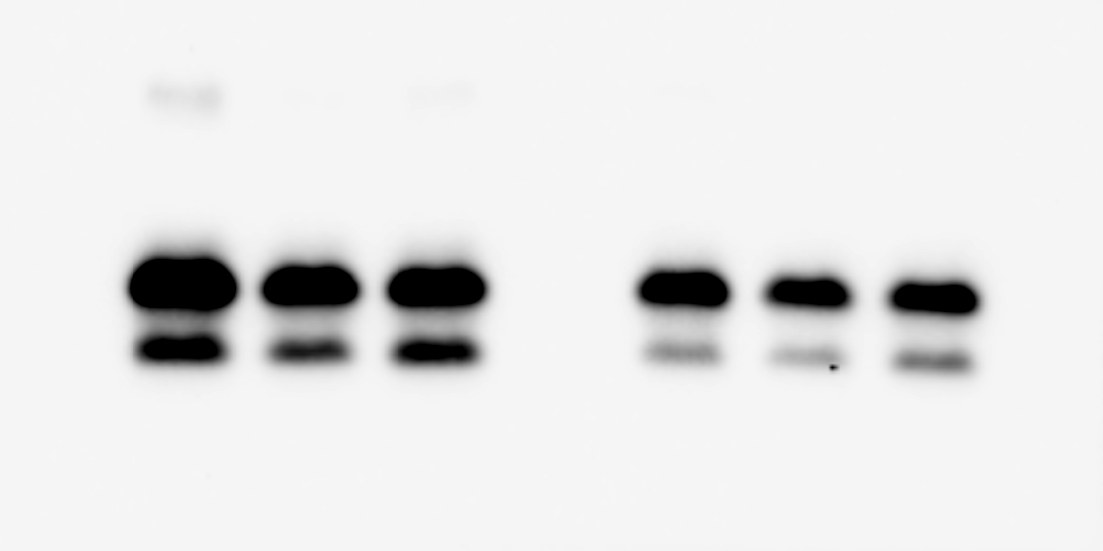

Supplement: Supplementary file 4 — Source data Fig. 2 [file 44321_2024_151_MOESM4_ESM.zip › EMM-2023-19183_SourceDataForFigure 2 /2B/LC3.tif]

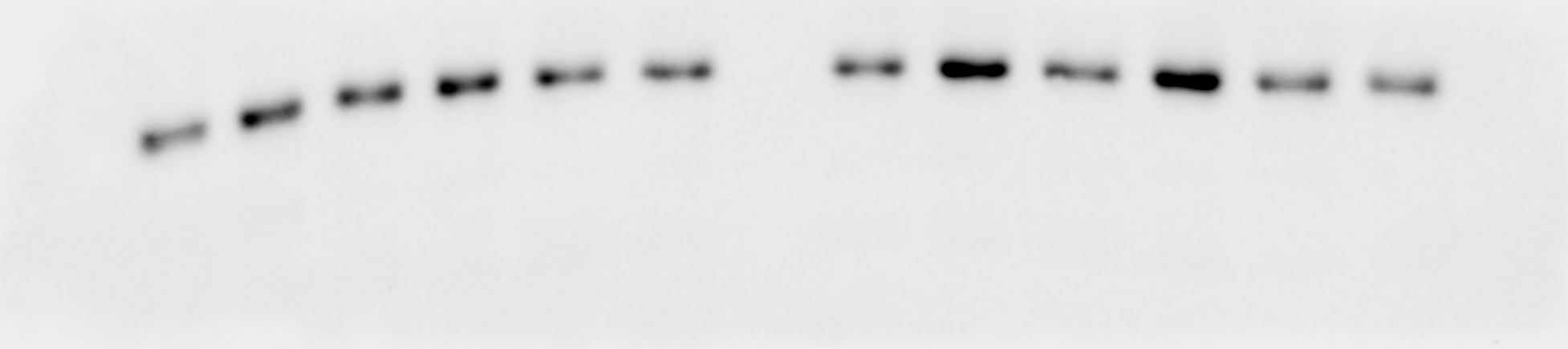

Supplement: Supplementary file 5 — Source data Fig. 3 [file 44321_2024_151_MOESM5_ESM.zip › EMM-2023-19183_SourceDataForFigure 3/3A/ATG3 uncropped.tif]

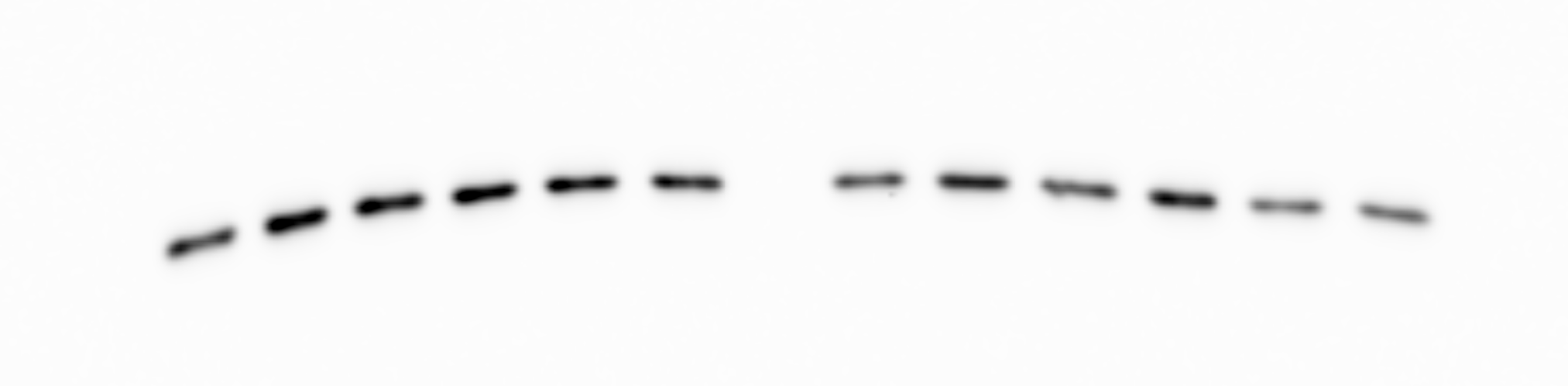

Supplement: Supplementary file 5 — Source data Fig. 3 [file 44321_2024_151_MOESM5_ESM.zip › EMM-2023-19183_SourceDataForFigure 3/3A/GAPDH of ATG3 uncropped.tif]

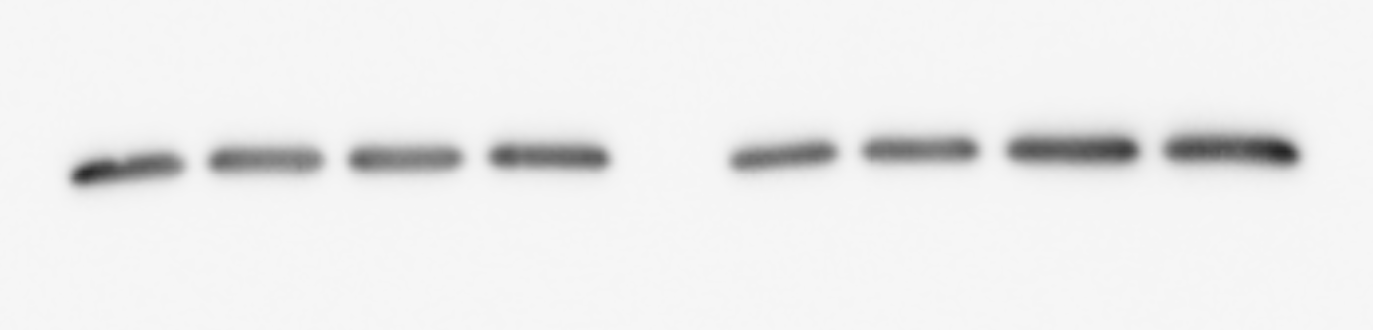

Supplement: Supplementary file 5 — Source data Fig. 3 [file 44321_2024_151_MOESM5_ESM.zip › EMM-2023-19183_SourceDataForFigure 3/3A/GAPDH of ATG16L1.tif]

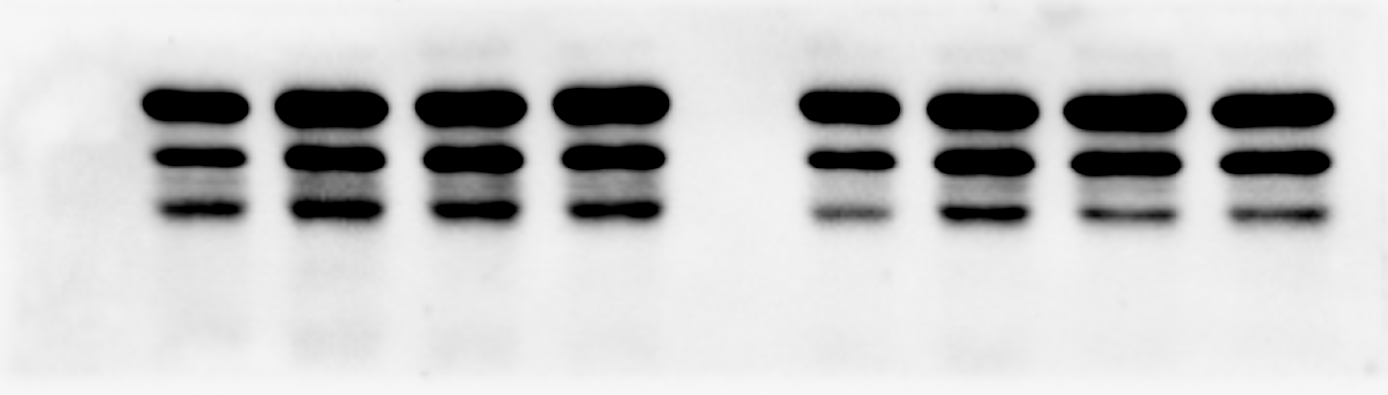

Supplement: Supplementary file 5 — Source data Fig. 3 [file 44321_2024_151_MOESM5_ESM.zip › EMM-2023-19183_SourceDataForFigure 3/3A/ATG16L1.tif]

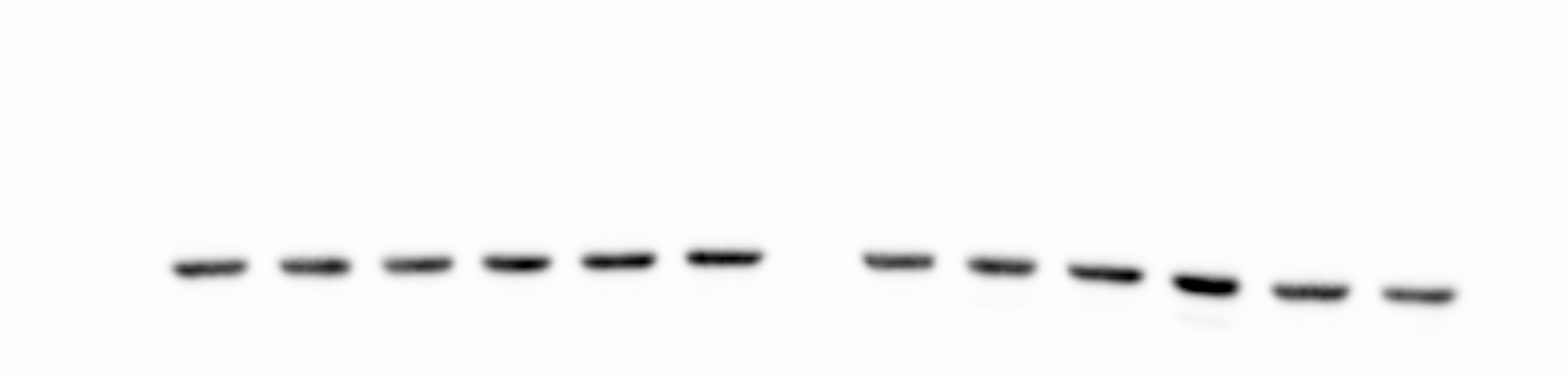

Supplement: Supplementary file 5 — Source data Fig. 3 [file 44321_2024_151_MOESM5_ESM.zip › EMM-2023-19183_SourceDataForFigure 3/3A/ATG5 uncropped.tif]

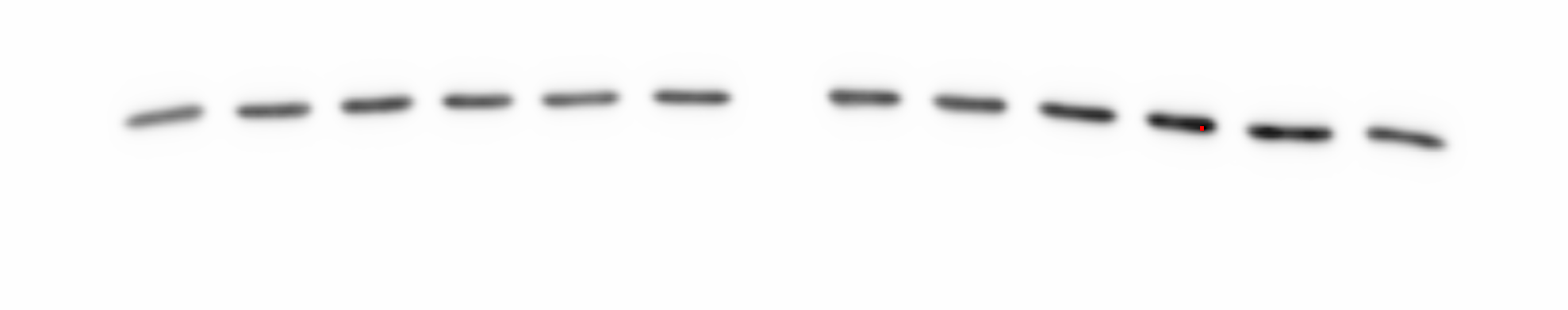

Supplement: Supplementary file 5 — Source data Fig. 3 [file 44321_2024_151_MOESM5_ESM.zip › EMM-2023-19183_SourceDataForFigure 3/3A/GAPDH of ATG5 uncropped.tif]

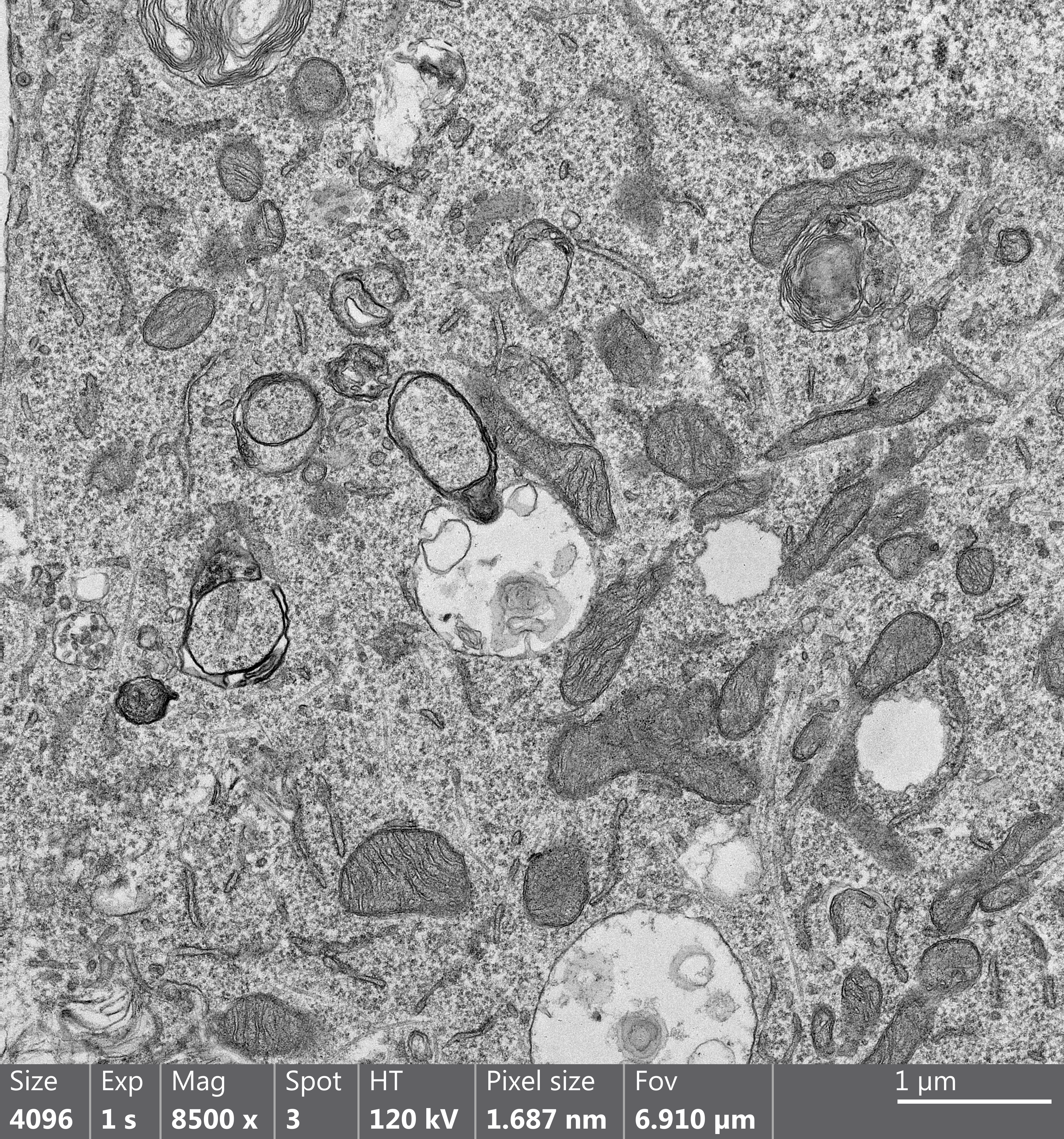

Supplement: Supplementary file 6 — Source data Fig. 4 [file 44321_2024_151_MOESM6_ESM.zip › EMM-2023-19183_SourceDataForFigure 4/4B/N-KO-treh 0014.tif]

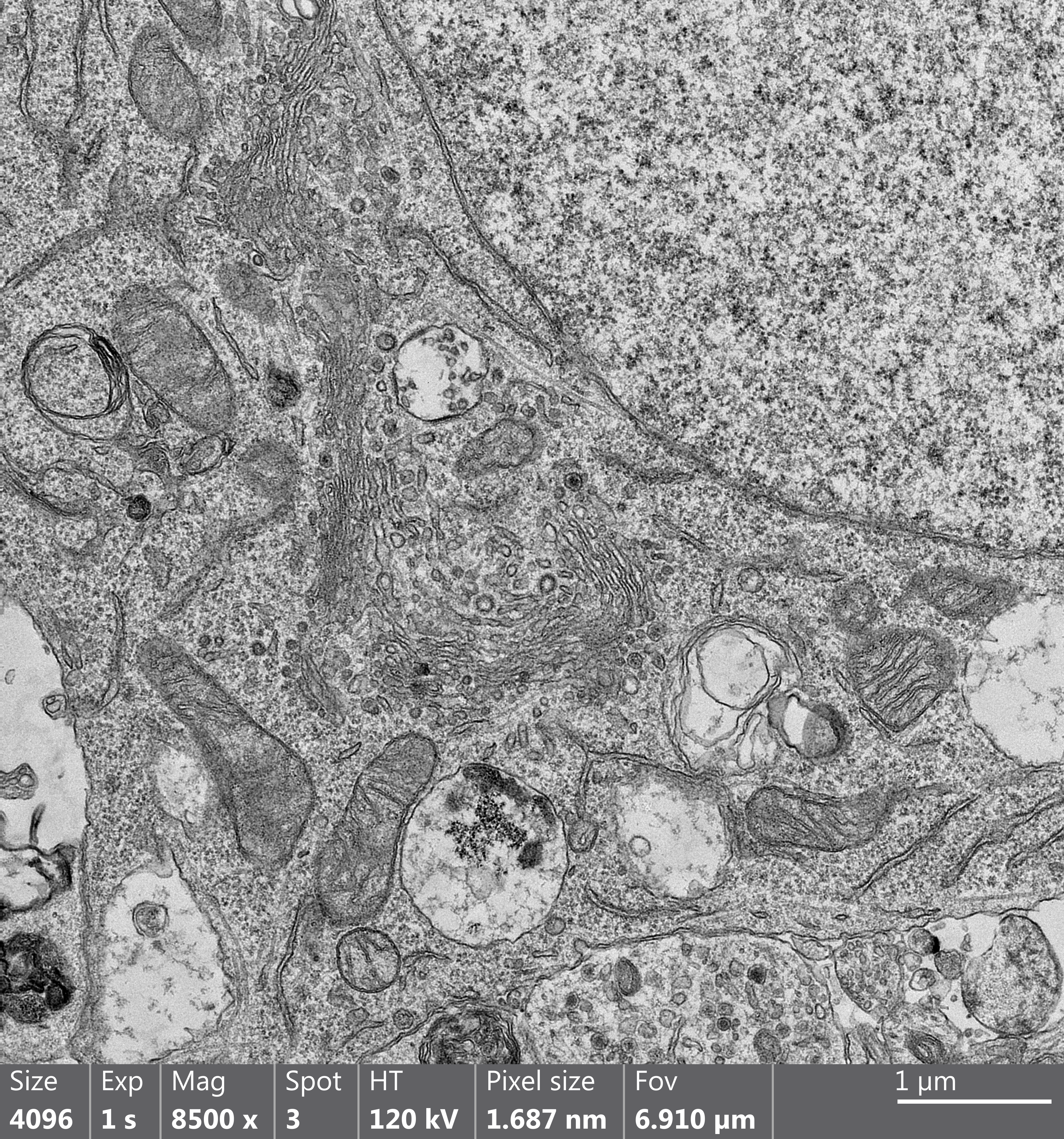

Supplement: Supplementary file 6 — Source data Fig. 4 [file 44321_2024_151_MOESM6_ESM.zip › EMM-2023-19183_SourceDataForFigure 4/4B/N-WT-treh 0015.tif]

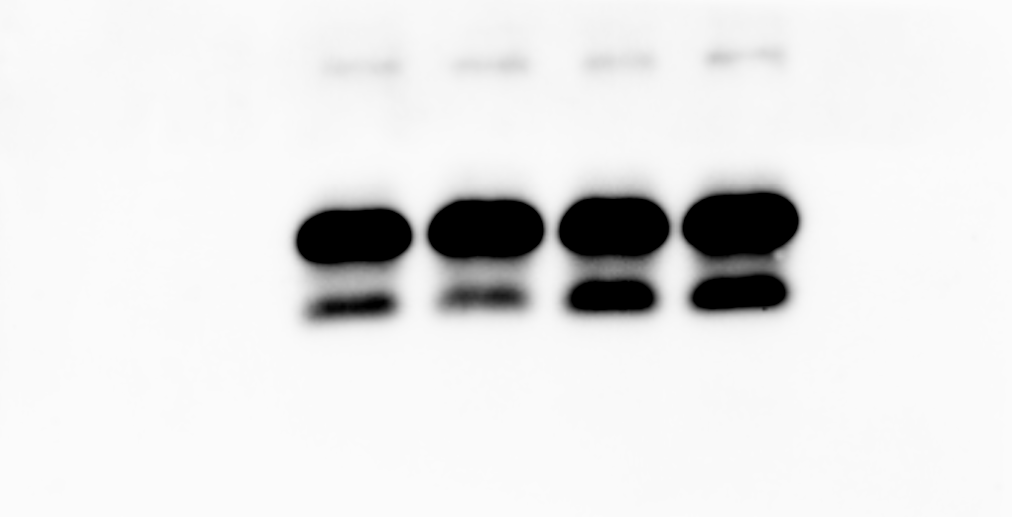

Supplement: Supplementary file 6 — Source data Fig. 4 [file 44321_2024_151_MOESM6_ESM.zip › EMM-2023-19183_SourceDataForFigure 4/4A/LC3.tif]

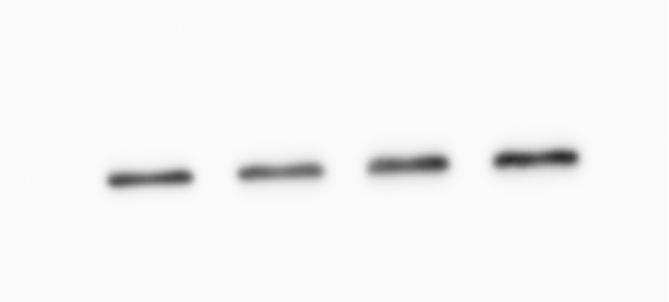

Supplement: Supplementary file 6 — Source data Fig. 4 [file 44321_2024_151_MOESM6_ESM.zip › EMM-2023-19183_SourceDataForFigure 4/4A/GAPDH.tif]

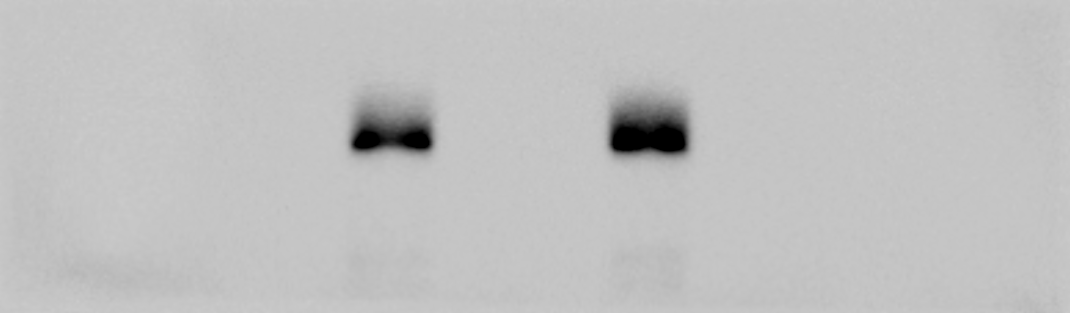

Supplement: Supplementary file 6 — Source data Fig. 4 [file 44321_2024_151_MOESM6_ESM.zip › EMM-2023-19183_SourceDataForFigure 4/4A/MeCP2.tif]

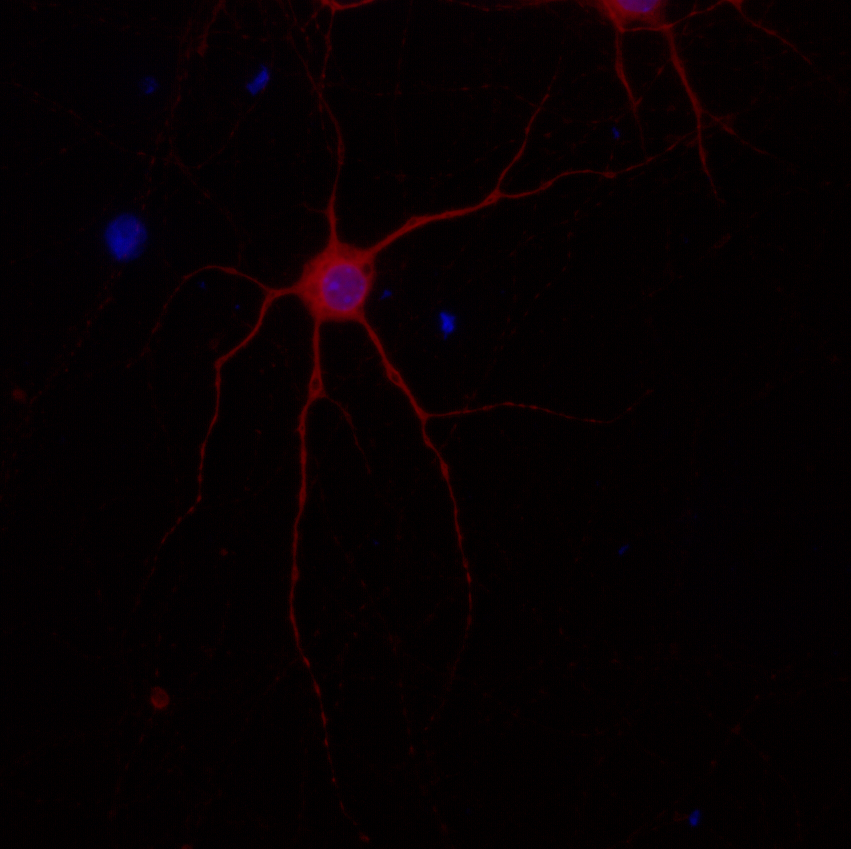

Supplement: Supplementary file 8 — Source data Fig. 6 [file 44321_2024_151_MOESM8_ESM.zip › EMM-2023-19183_SourceDataForFigure 6/6A/KO Trehalose 8.tif]

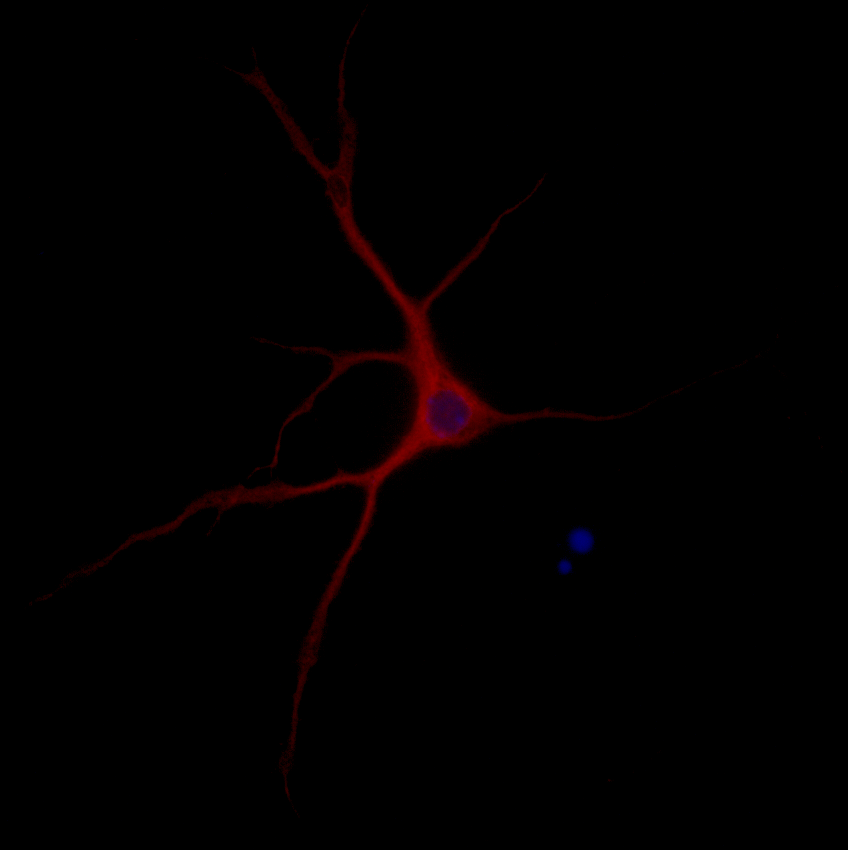

Supplement: Supplementary file 8 — Source data Fig. 6 [file 44321_2024_151_MOESM8_ESM.zip › EMM-2023-19183_SourceDataForFigure 6/6A/WT Untreated 3.tif]

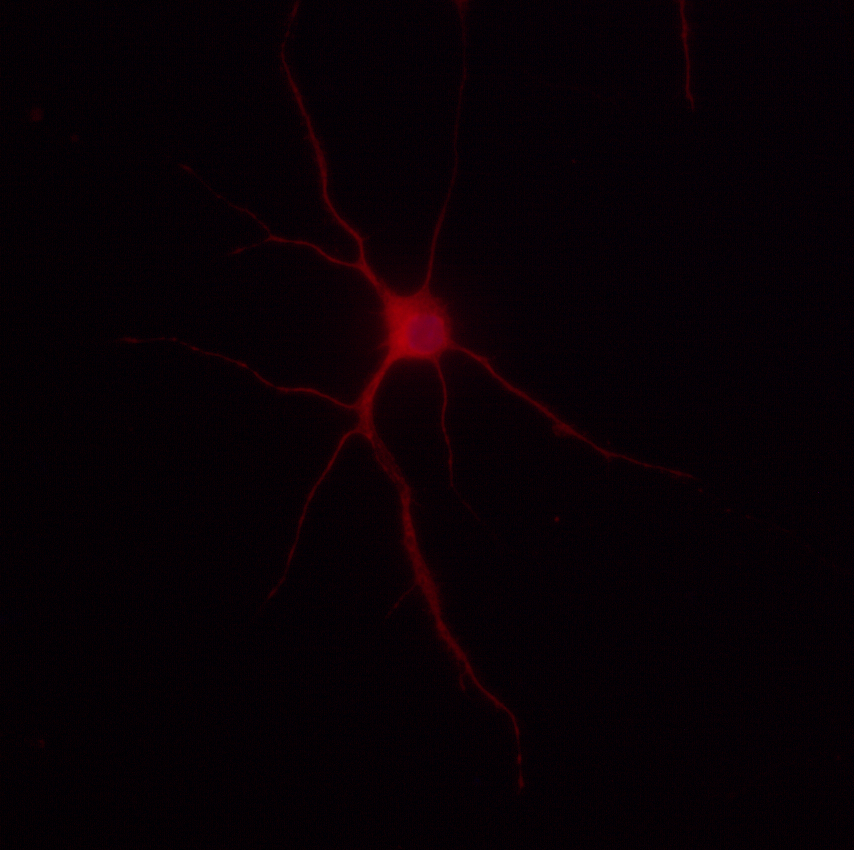

Supplement: Supplementary file 8 — Source data Fig. 6 [file 44321_2024_151_MOESM8_ESM.zip › EMM-2023-19183_SourceDataForFigure 6/6A/WT Trehalose 10.tif]

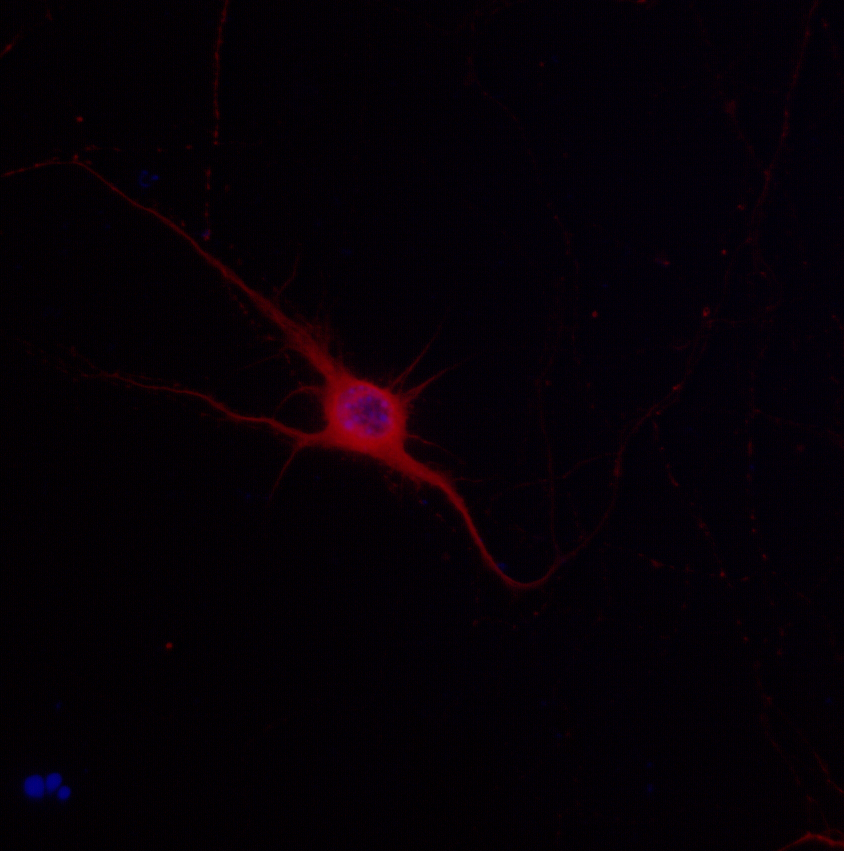

Supplement: Supplementary file 8 — Source data Fig. 6 [file 44321_2024_151_MOESM8_ESM.zip › EMM-2023-19183_SourceDataForFigure 6/6A/KO Untreated 30.tif]

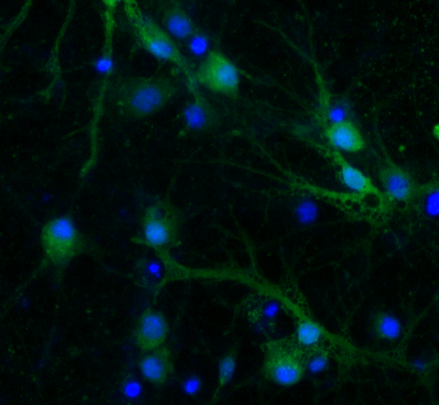

Supplement: Supplementary file 8 — Source data Fig. 6 [file 44321_2024_151_MOESM8_ESM.zip › EMM-2023-19183_SourceDataForFigure 6/6B/KO treh basal (E10)(RGB)-roi.tif]

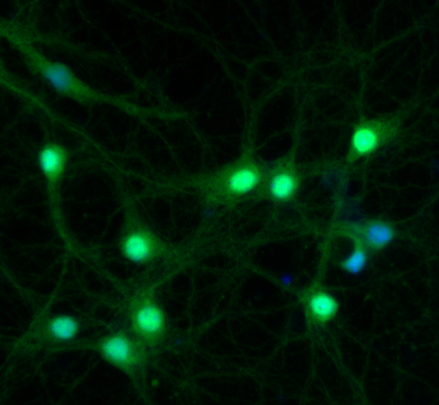

Supplement: Supplementary file 8 — Source data Fig. 6 [file 44321_2024_151_MOESM8_ESM.zip › EMM-2023-19183_SourceDataForFigure 6/6B/WT treh NMDA (B09) (RGB)-roi.tif]

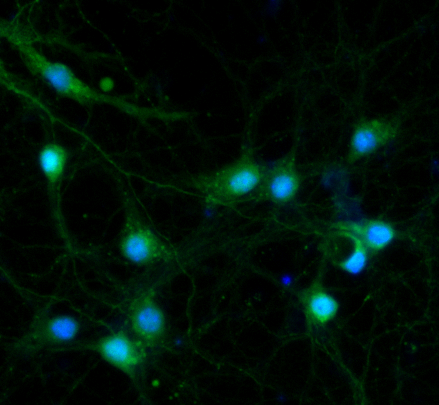

Supplement: Supplementary file 8 — Source data Fig. 6 [file 44321_2024_151_MOESM8_ESM.zip › EMM-2023-19183_SourceDataForFigure 6/6B/WT treh basal (B09) (RGB)-roi.tif]

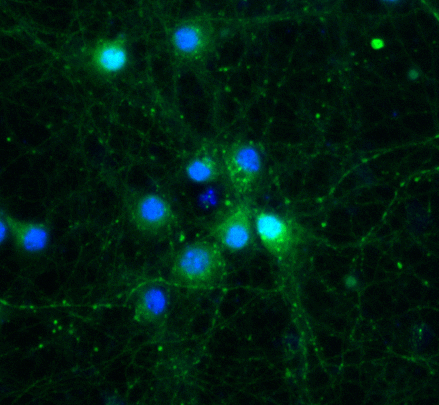

Supplement: Supplementary file 8 — Source data Fig. 6 [file 44321_2024_151_MOESM8_ESM.zip › EMM-2023-19183_SourceDataForFigure 6/6B/WT untreated basale (F02)-roi .tif]

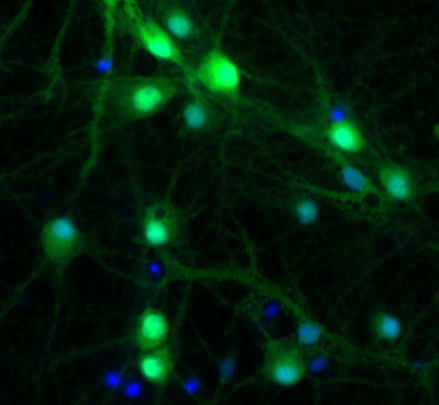

Supplement: Supplementary file 8 — Source data Fig. 6 [file 44321_2024_151_MOESM8_ESM.zip › EMM-2023-19183_SourceDataForFigure 6/6B/KO treh NMDA (E10) (RGB)-roi.tif]

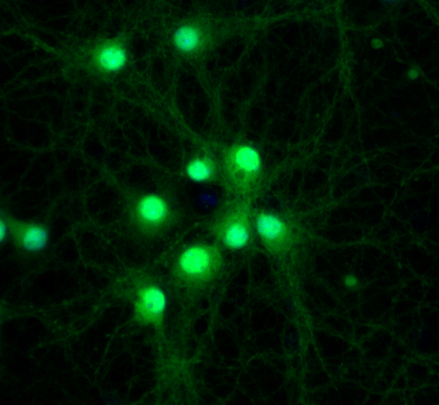

Supplement: Supplementary file 8 — Source data Fig. 6 [file 44321_2024_151_MOESM8_ESM.zip › EMM-2023-19183_SourceDataForFigure 6/6B/WT untreated NMDA (F02) (RGB)-roi.tif]

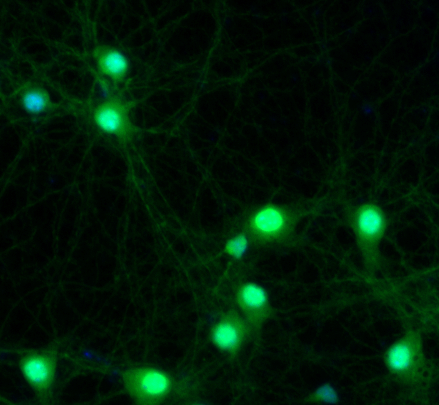

Supplement: Supplementary file 8 — Source data Fig. 6 [file 44321_2024_151_MOESM8_ESM.zip › EMM-2023-19183_SourceDataForFigure 6/6B/KO untreated NMDA (C03)-1.tif (RGB)-roi.tif]

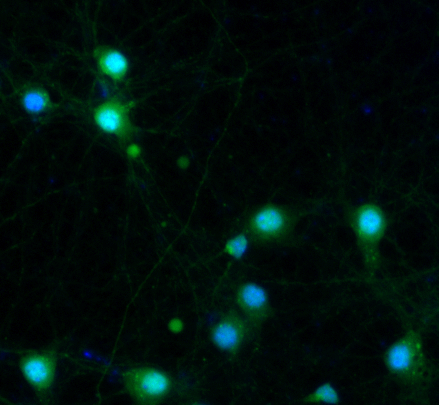

Supplement: Supplementary file 8 — Source data Fig. 6 [file 44321_2024_151_MOESM8_ESM.zip › EMM-2023-19183_SourceDataForFigure 6/6B/KO untreated basal (C03)-1.tif (RGB)-roi.tif]

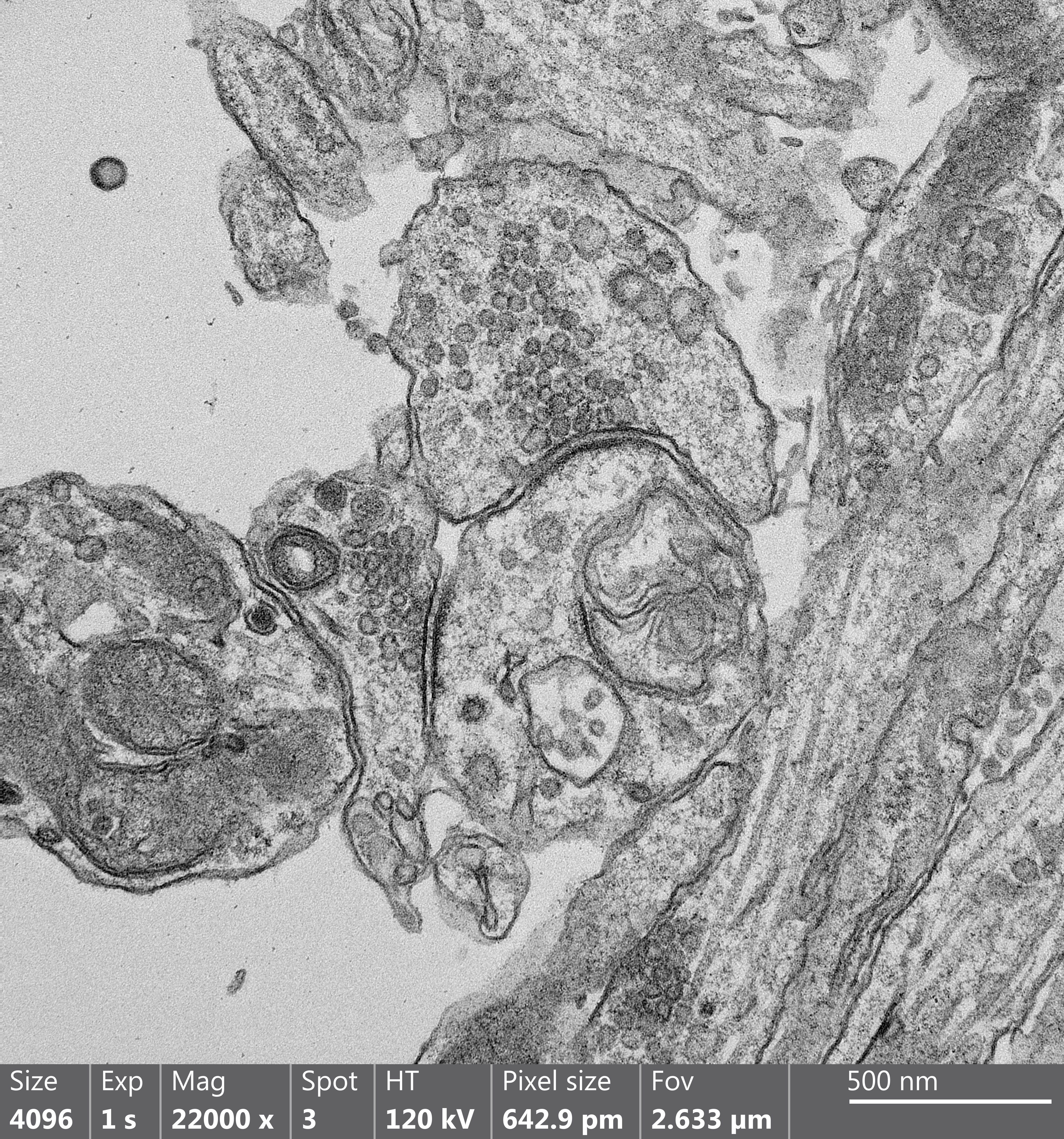

Supplement: Supplementary file 8 — Source data Fig. 6 [file 44321_2024_151_MOESM8_ESM.zip › EMM-2023-19183_SourceDataForFigure 6/6C/Syn_WT-TREH 0005.tif]

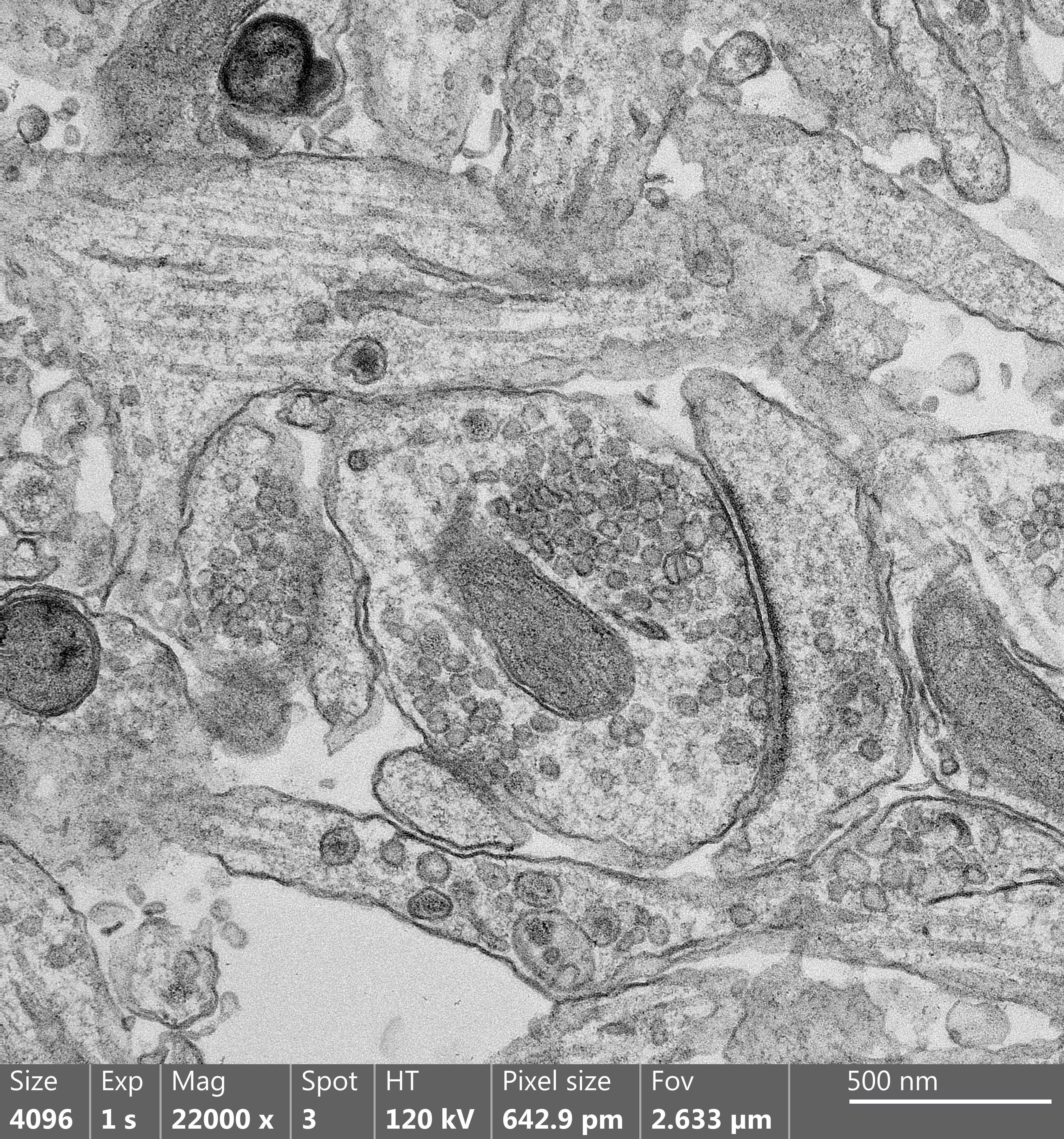

Supplement: Supplementary file 8 — Source data Fig. 6 [file 44321_2024_151_MOESM8_ESM.zip › EMM-2023-19183_SourceDataForFigure 6/6C/Syn_KO 0015.tif]

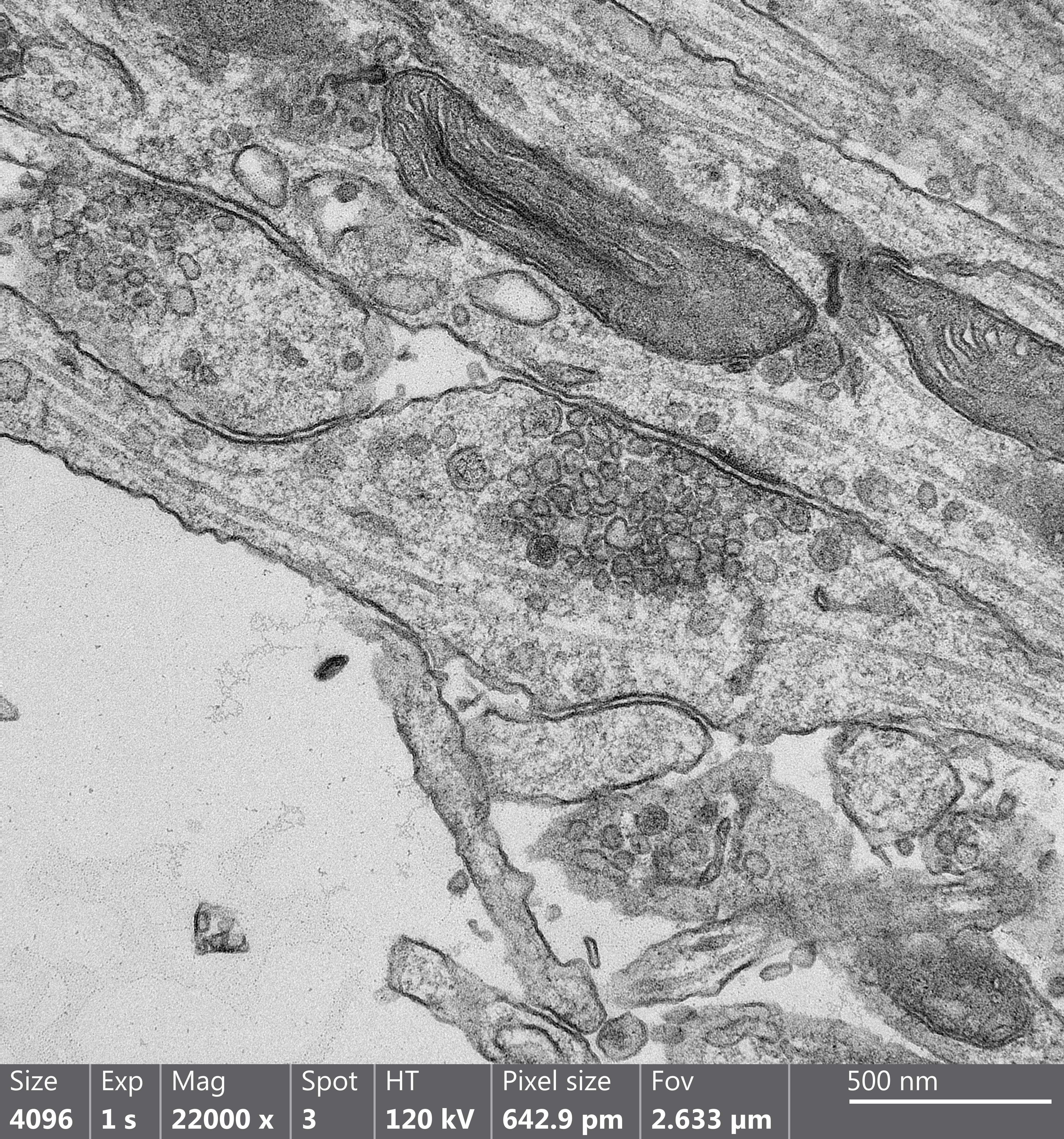

Supplement: Supplementary file 8 — Source data Fig. 6 [file 44321_2024_151_MOESM8_ESM.zip › EMM-2023-19183_SourceDataForFigure 6/6C/Syn_KO-TREH 0001.tif]

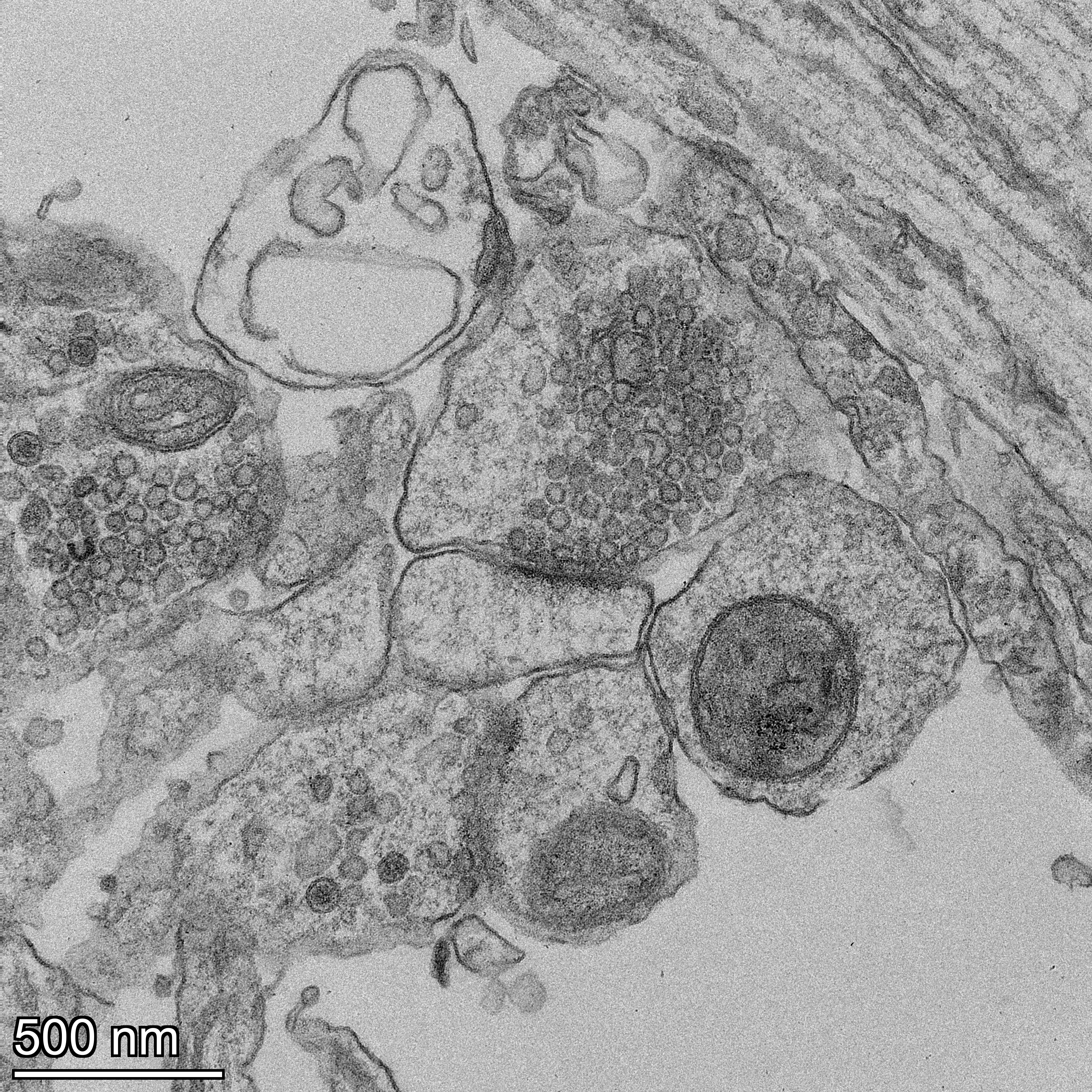

Supplement: Supplementary file 8 — Source data Fig. 6 [file 44321_2024_151_MOESM8_ESM.zip › EMM-2023-19183_SourceDataForFigure 6/6C/Syn_WT 0023.tif]
