# Supplementary material for: Unraveling autophagic imbalances and therapeutic insights in Mecp2-deficient models
Source: EMBO Mol Med. 2024 Oct 14;16(11):2795–826. doi: 10.1038/s44321-024-00151-w (PMC11555085; doi:10.1038/s44321-024-00151-w)
Supplement: Supplementary file 5 — Source data Fig. 3 [file 44321_2024_151_MOESM5_ESM.zip › EMM-2023-19183_SourceDataForFigure 3/3B 3C Suppl4B/README.rtf]

Raw peak intensity of LC-MS signals.To obtain Volcano plots and box plots, data were normalized as described in Materials and Methods section.Rows: lipids identified based on MassHunter Lipid AnnotatorColumns: sample name
