# Supplementary material for: Unraveling autophagic imbalances and therapeutic insights in Mecp2-deficient models
Source: EMBO Mol Med. 2024 Oct 14;16(11):2795–826. doi: 10.1038/s44321-024-00151-w (PMC11555085; doi:10.1038/s44321-024-00151-w)
Supplement: Supplementary file 10 — Expanded View Figures [file 44321_2024_151_MOESM10_ESM.pdf]

Expanded View Figures

Figure EV1. Mecp2 deficiency leads to a defective autophagosome maturation.

(**A**) Representative western blot from lysates of WT and KO cortical neurons at different days in culture (DIV). LC3B-I and LC3B-II intensities were quantified by densitometric analysis. LC3B-II/LC3B-I ratio was calculated. Mecp2 signal is shown as a genotype control. Data were expressed as median ± min/max ($n = 5$ embryos from three independent experiments). Mann–Whitney test, *$p < 0.05$ (3 DIV $p = 0.8413$; 7 DIV $p > 0.99$; 14 DIV $p = 0.0159$; 21 DIV $p = 0.0556$). (**B**) Western blot from lysates of WT and KO cortical neurons at different days in culture (DIV). NeuN, PSD-95, SNAP25, and VAMP2 signals are shown as markers of developmental progression ($n = 1$). (**C**) Representative TEM micrographs of WT and KO cortical neurons (14 DIV) under resting conditions showing examples of autophagic vacuoles in WT neurons, and the presence of immature autophagic structures in neuronal processes in KO neurons. (**D**) Representative western blot from lysates of WT and KO cortical neurons (14 DIV). VGLUT1 intensity was quantified by densitometric analysis and normalized on GAPDH intensity. Data were expressed as median ± min/max, normalized on WT ($n = 6$ embryos from three independent experiments). Mann–Whitney test, **$p < 0.01$ ($p = 0.0022$).

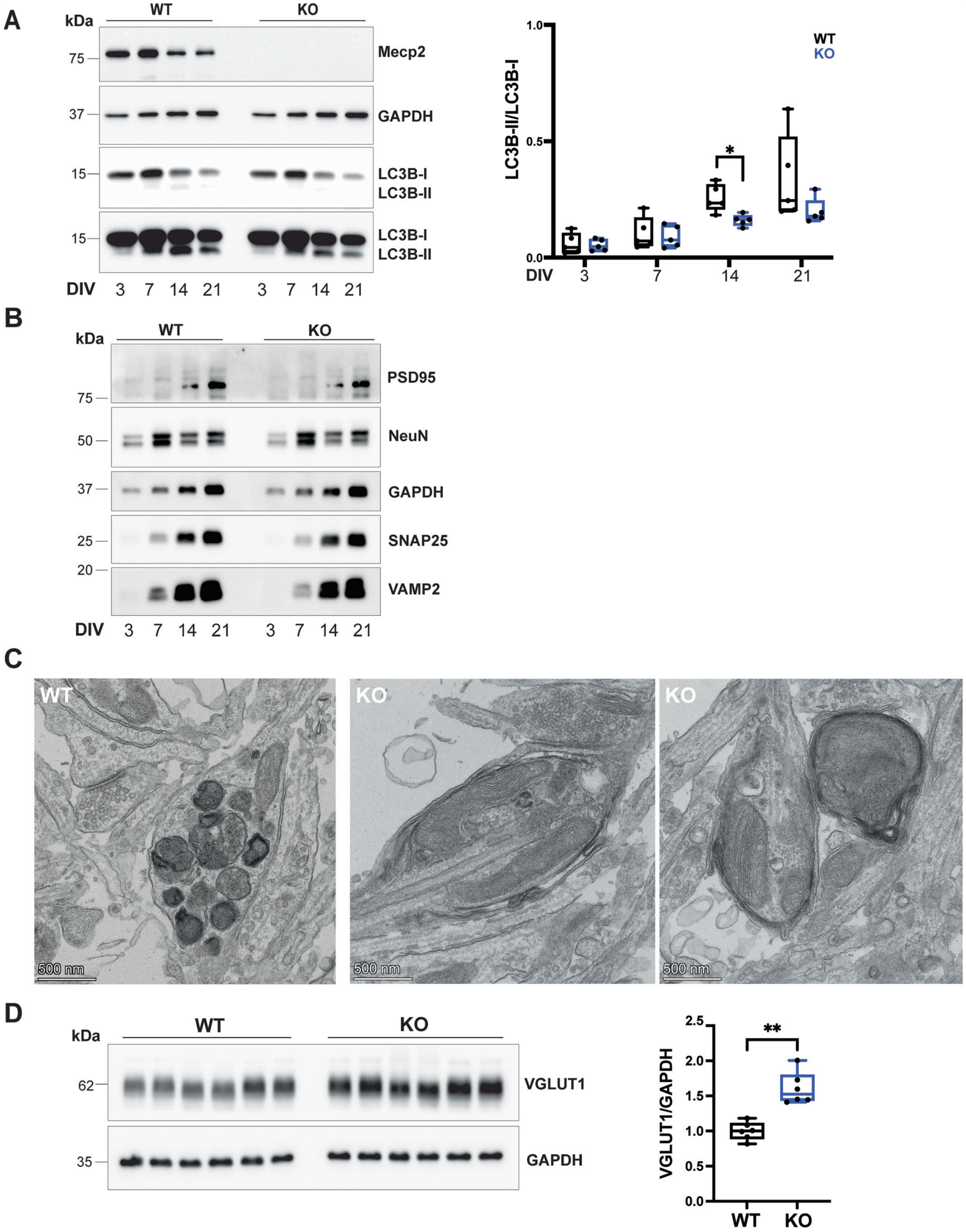
A
kDa
WT
KO
75
37
15
15
Mecp2
GAPDH
LC3B-I
LC3B-II
LC3B-I
LC3B-II
DIV 3 7 14 21 3 7 14 21
LC3B-II/LC3B-I
1.0
0.5
0.0
WT
KO
*
DIV 3 7 14 21
B
kDa
WT
KO
75
50
37
25
20
PSD95
NeuN
GAPDH
SNAP25
VAMP2
DIV 3 7 14 21 3 7 14 21
C
WT
KO
KO
500 nm
500 nm
500 nm
D
kDa
WT
KO
62
35
VGLUT1
GAPDH
VGLUT1/GAPDH
2.5
2.0
1.5
1.0
0.5
0.0
**
WT KO

 © The Author(s)

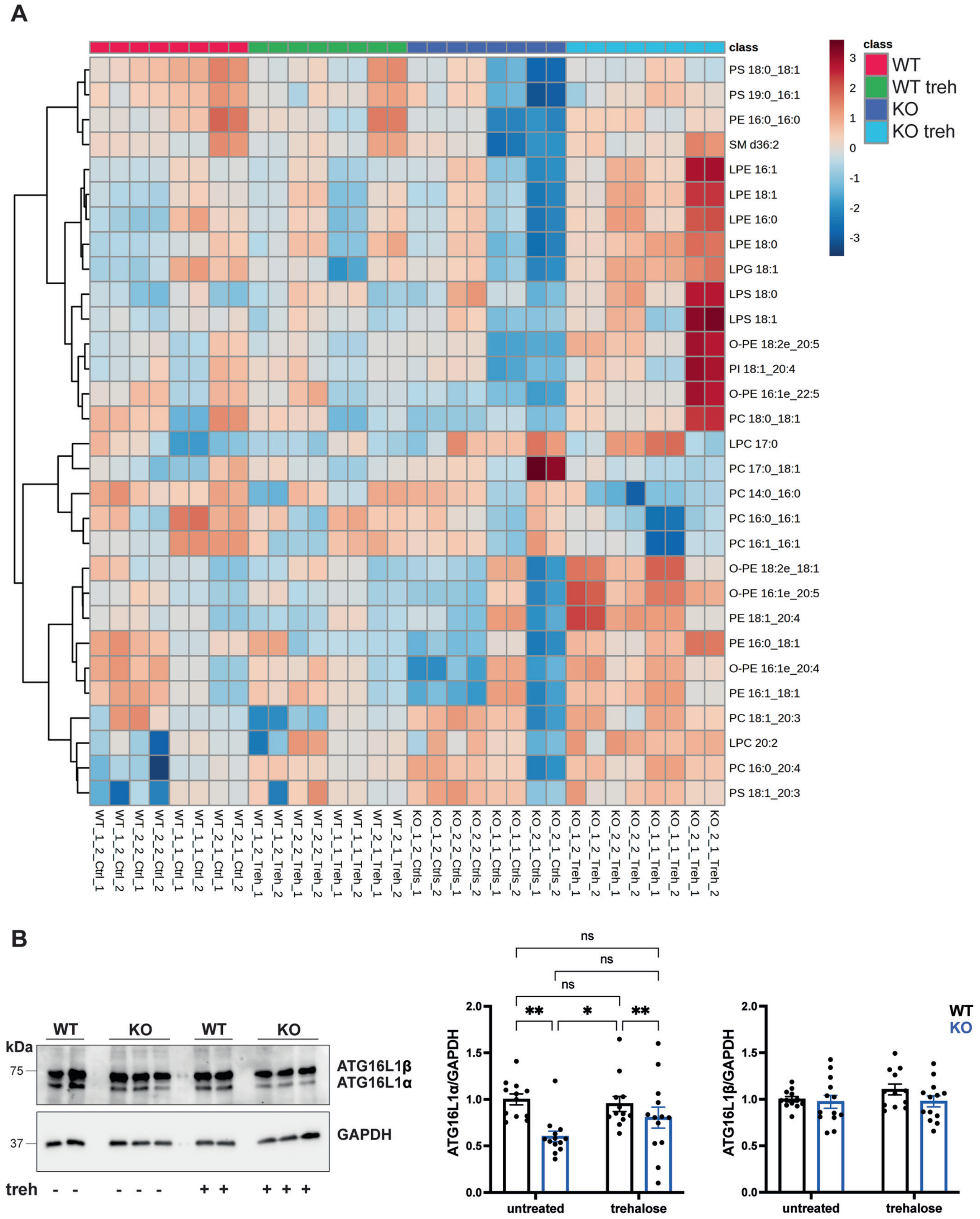
A
class
PS 18:0_18:1
PS 19:0_16:1
PE 16:0_16:0
SM d36:2
LPE 16:1
LPE 18:1
LPE 16:0
LPE 18:0
LPG 18:1
LPS 18:0
LPS 18:1
O-PE 18:2e_20:5
PI 18:1_20:4
O-PE 16:1e_22:5
PC 18:0_18:1
LPC 17:0
PC 17:0_18:1
PC 14:0_16:0
PC 16:0_16:1
PC 16:1_16:1
O-PE 18:2e_18:1
O-PE 16:1e_20:5
PE 18:1_20:4
PE 16:0_18:1
O-PE 16:1e_20:4
PE 16:1_18:1
PC 18:1_20:3
LPC 20:2
PC 16:0_20:4
PS 18:1_20:3
WT_1_2_Ctrl_1
WT_1_2_Ctrl_2
WT_2_2_Ctrl_1
WT_2_2_Ctrl_2
WT_1_1_Ctrl_1
WT_1_1_Ctrl_2
WT_2_1_Ctrl_1
WT_2_1_Ctrl_2
WT_1_2_Treh_1
WT_1_2_Treh_2
WT_2_2_Treh_1
WT_2_2_Treh_2
WT_1_1_Treh_1
WT_1_1_Treh_2
WT_2_1_Treh_1
WT_2_1_Treh_2
KO_1_2_Ctrls_1
KO_1_2_Ctrls_2
KO_2_2_Ctrls_1
KO_2_2_Ctrls_2
KO_1_1_Ctrls_1
KO_1_1_Ctrls_2
KO_2_1_Ctrls_1
KO_2_1_Ctrls_2
KO_1_2_Treh_1
KO_1_2_Treh_2
KO_2_2_Treh_1
KO_2_2_Treh_2
KO_1_1_Treh_1
KO_1_1_Treh_2
KO_2_1_Treh_1
KO_2_1_Treh_2
3
2
1
0
-1
-2
-3
class
WT
WT treh
KO
KO treh
B
WT
KO
WT
KO
kDa
75
37
ATG16L1β
ATG16L1α
GAPDH
treh - - - - - + + + + +
ns
ns
ns
** * **
ATG16L1α/GAPDH
2.0
1.5
1.0
0.5
0.0
untreated
trehalose
ATG16L1β/GAPDH
2.0
1.5
1.0
0.5
0.0
untreated
trehalose
WT
KO

© The Author(s)

Figure EV2. ATG16L1 and lipid content of WT and KO treated and untreated neurons.

(**A**) Heatmap obtained using the 30 most significant lipids in the complete dataset (ANOVA test). Clustering distance measure: Euclidean. Clustering method for lipids was Ward. PE: diacylglycerophosphoethanolamines; O-PE: alkyl, acylglycerophosphoethanolamines; PS: diacylglycerophosphoserines; PI: diacylglycerophosphoinositols; PC: diacylglycerophosphocholines; SM: ceramide phosphocholines (sphingomyelins); LPG: monoacylglycerophosphoglycerols; LPE: diacylglycerophosphoethanolamines; LPS: monoacylglycerophosphoserines; LPC: monoacylglycerophosphocholines. (**B**) Representative western blot from lysates of WT and KO cortical neurons (14 DIV) under resting condition or incubated with 25 mM trehalose for 48 h. ATG16L1α and ATG16L1β intensies were quantified by densitometric analysis and normalized on GAPDH intensity. Data were mean ± SEM, normalized on WT ($n = 12$–13 embryos from five independent experiments). Two-way ANOVA with Tukey's multiple comparisons test, *$p < 0.05$, **$p < 0.01$ (ATG16L1α: ut WT vs ut KO $p = 0.0092$, ut WT vs treh WT $p = 0.7545$, ut WT vs treh KO $p = 0.3637$, ut KO vs treh WT $p = 0.0191$, ut KO vs treh KO $p = 0.7545$, treh WT vs treh KO $p = 0.0092$).

 © The Author(s)

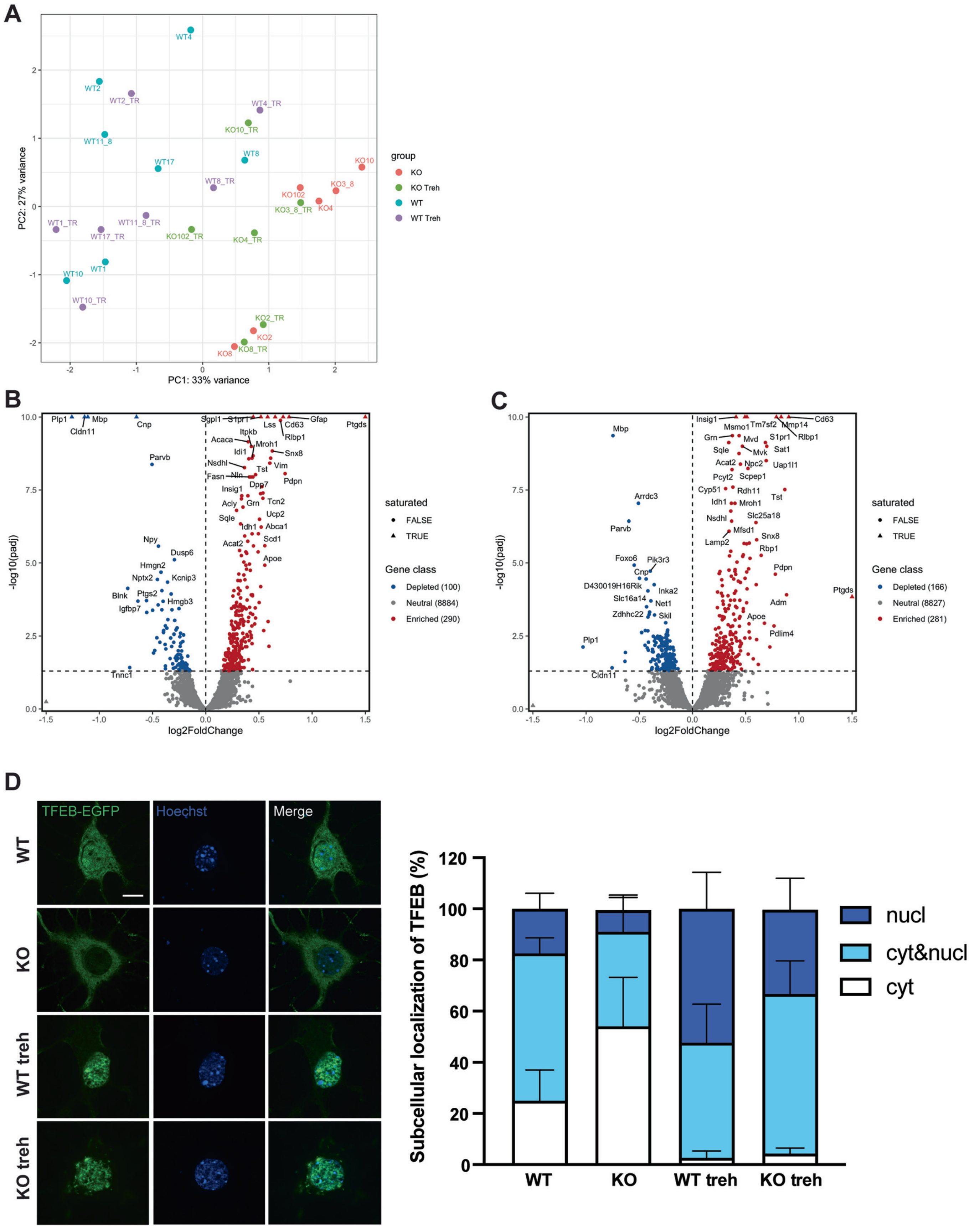
A
PC1: 33% variance
PC2: 27% variance
group
KO
KO Treh
WT
WT Treh
B
C
log2FoldChange
-log10(padj)
saturated
FALSE
TRUE
Gene class
Depleted (100)
Neutral (8884)
Enriched (290)
Depleted (166)
Neutral (8827)
Enriched (281)
D
TFEB-EGFP
Hoechst
Merge
WT
KO
WT treh
KO treh
Subcellular localization of TFEB (%)
nucl
cyt&nucl
cyt

Figure EV3. Trehalose administration induces transcriptional changes related to lipidic and auto-lysosomal pathways along with TFEB nuclear translocation.

(**A**) Principal component analysis (PCA) plot of WT and KO neurons untreated or treated with trehalose. Percentage of variance is reported for both PC1 (first component) and PC2 (second component). (**B, C**) Volcano plot showing the upregulated (red dots) and downregulated (blue dots) DEGs of WT treh vs WT (left) and KO treh vs KO (right) neurons. The x-axis represents the log2 fold change (FC), while the y-axis is the −log 10 (P adj) of RNA-seq data from eight independent biological replicates. The *p* value was computed using DESeq2 (see Methods). The adjusted *p* value is obtained using the Benjamini–Hochberg method for correction for multiple hypotheses testing. Differentially expressed genes were assessed using the adjusted *p* value threshold of 0.05. (**D**) Representative confocal images of WT and KO cortical neurons (14 DIV) transfected with a TFEB-EGFP plasmid, under resting conditions or incubated with 25 mM trehalose for 48 h (treh). GFP immunolabelling and Hoechst nuclear stain are shown. Results are expressed as percentage of cells assigned to each subcellular localization category (either only nuclear, only cytosolic, or nuclear + cytosolic), mean ± SEM ($n > 10$ cells analyzed/condition for each experiment, from 3 to 4 independent experiments); Scale bar: 10 µm.

 © The Author(s)

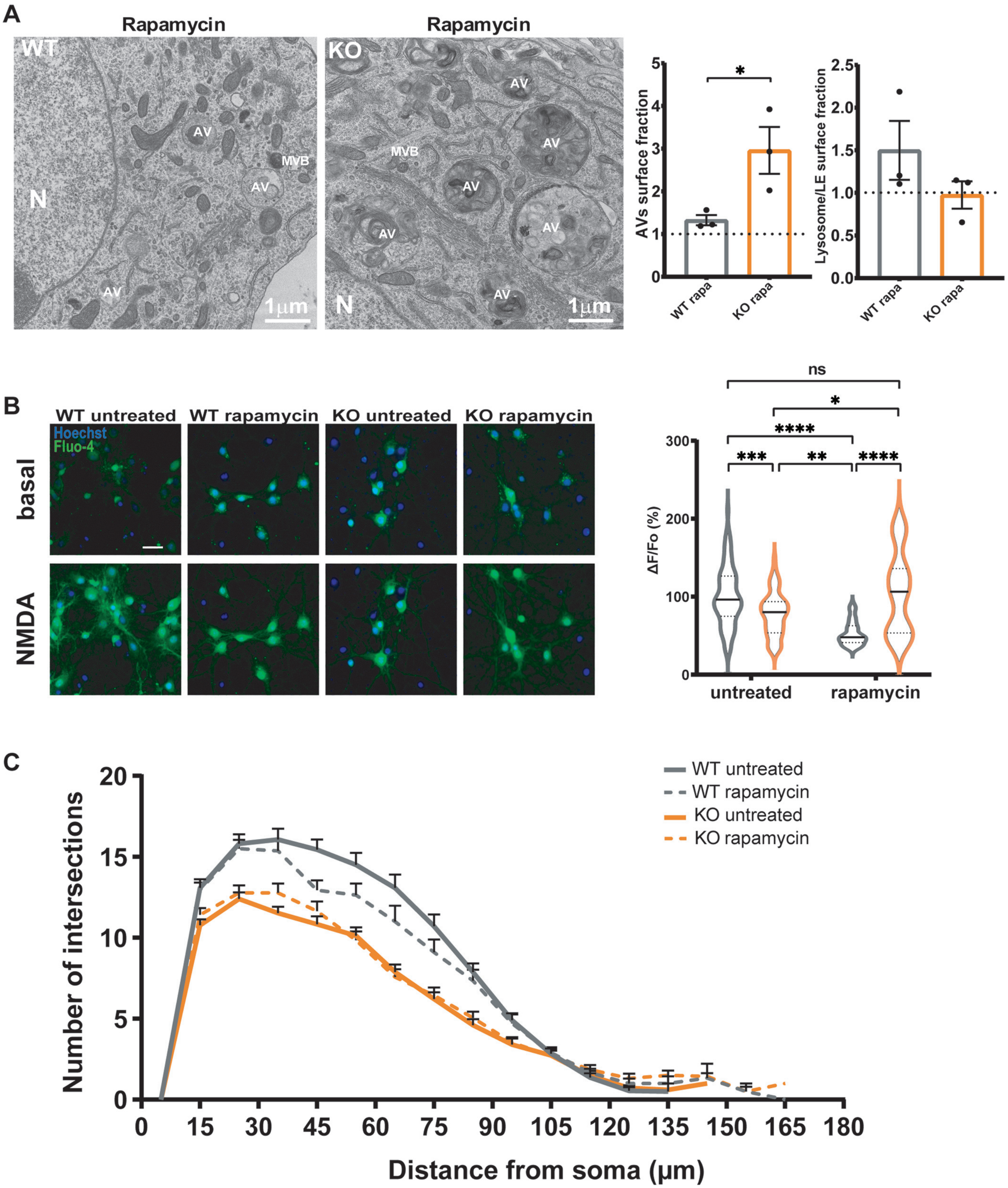
A
Rapamycin
Rapamycin
WT
KO
AV
MVB
N
1μm
AVs surface fraction
Lysosome/LE surface fraction
WT rapa
KO rapa
B
WT untreated
WT rapamycin
KO untreated
KO rapamycin
Hoechst
Fluo-4
basal
NMDA
ΔF/Fo (%)
untreated
rapamycin
ns
C
Number of intersections
Distance from soma (μm)
WT untreated
WT rapamycin
KO untreated
KO rapamycin

Figure EV4. Rapamycin enhances autophagic vacuole content and neuronal activity in Mecp2 KO neurons.

(**A**) Representative TEM micrographs of WT and KO cortical neurons (14 DIV) incubated with 100 nM rapamycin for 48 h. The area occupied by autophagosomes and autolysosomes (AVs) or by lysosomes over the total cell area in the image was quantified. Data were expressed as mean ± SEM normalized on WT untreated (dashed line on the graph) ($n = 3$ independent experiments, with 58 WT and 61 KO rapa-treated neurons analyzed). N nucleus, AV autophagosome, *=enlarged lysosomes. Unpaired *T*-test, *$p < 0.05$ (Avs surface fraction: rapa WT vs rapa KO $p = 0.0437$; Lysosome/ LE surface fraction: WT rapa vs KO rapa $p = 0.2410$). (**B**) Representative images of WT and KO primary cortical neurons (14 DIV) loaded with Fluo-4 and exposed to 100 μM NMDA. Scale bar: 40 μm. Data indicate the Fluo-4 intensity (ΔF/F0) of WT and KO treated neurons (violin plots, median ± quartiles; $n = 105$ cells for WT untreated, $n = 32$ cells for WT rapamycin, $n = 63$ cells for KO untreated and $n = 20$ cells for KO rapamycin). Two-way ANOVA followed by Tukey's multiple comparisons test *$p < 0.05$, **$p < 0.01$, ***$p < 0.001$, ****$p < 0.0001$ (ut WT vs ut KO $p = 0.0004$, ut WT vs rapa WT $p < 0.0001$, ut WT vs rapa KO $p = 0.9243$, ut KO vs rapa WT $p = 0.0052$, ut KO vs rapa KO $p = 0.0116$, rapa WT vs rapa KO $p < 0.0001$). (**C**) The graph reports the average number of intersections measured by Sholl analysis every 15 μm from the soma of WT untreated ($N = 30$ from 2 embryos) or treated ($N = 48$ from 2 embryos) and KO untreated ($N = 30$ from 2 embryos) or treated ($N = 48$ from 2 embryos) neurons. Error bars indicate ±SEM.

 © The Author(s)

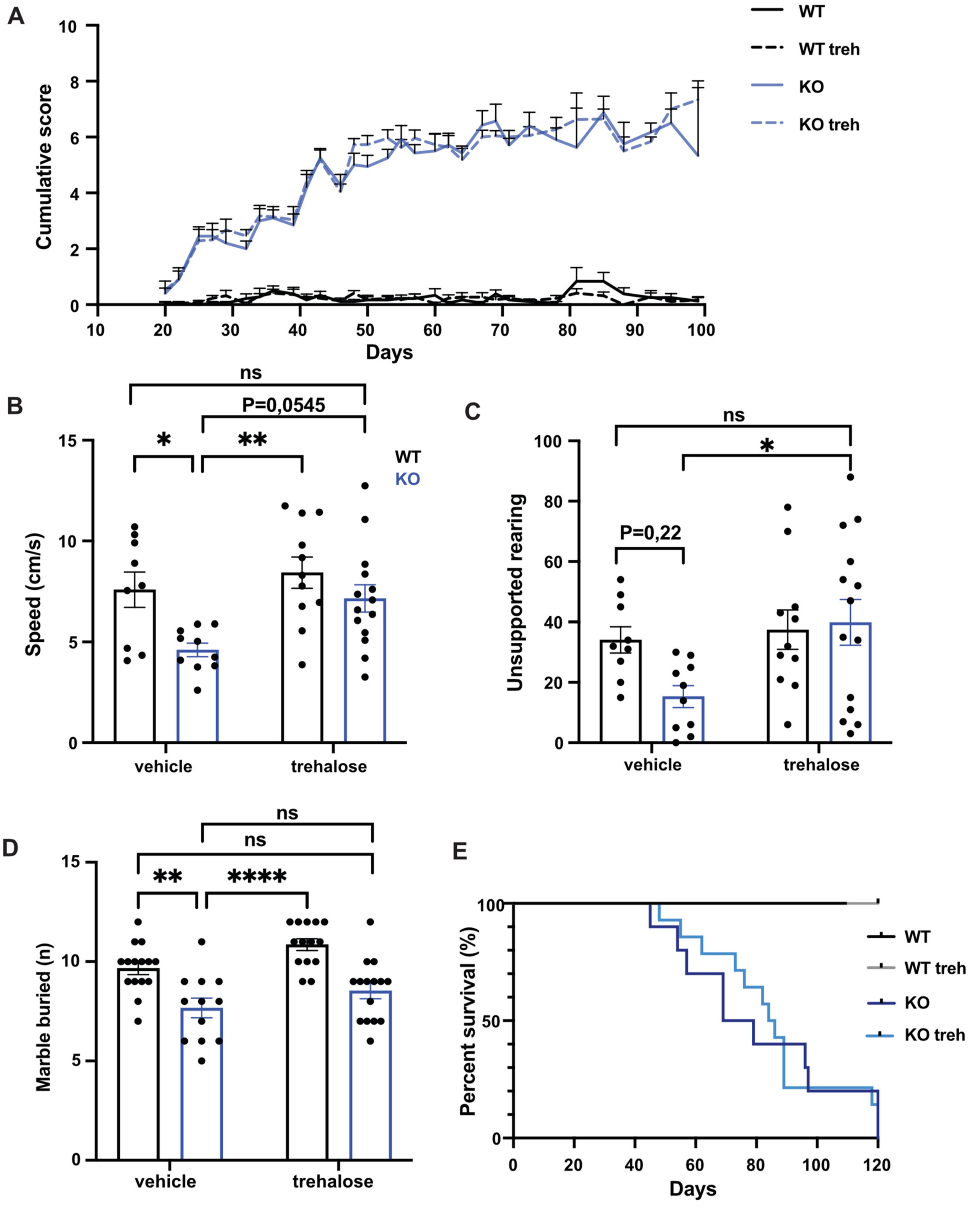
A
WT
WT treh
KO
KO treh
Cumulative score
Days
B
ns
P=0,0545
*
**
WT
KO
Speed (cm/s)
vehicle
trehalose
C
ns
*
P=0,22
Unsupported rearing
vehicle
trehalose
D
ns
ns
**
****
Marble buried (n)
vehicle
trehalose
E
WT
WT treh
KO
KO treh
Percent survival (%)
Days

Figure EV5. Behavioral tests and lifespan analysis in WT and *Mecp2* KO mice upon trehalose treatment.

(**A**) Cumulative score. (**B**) Speed. (**C**) Unsupported rearing. (**D**) Marble burying assay. (**E**) Kaplan–Mayer survival curve of the four experimental groups: WT untreated ($N = 9$) or treated ($N = 11$), and Mecp2-KO untreated ($N = 10$) or treated ($N = 14$). For Marble analysis, $n = 15$ WT vehicle, 12 = KO vehicle, 14 = WT trehalose, 15 = KO trehalose. Bars represent mean values with ± SEM. Behavioral data for **B–D** were analyzed using two-way ANOVA followed by Tukey's multiple comparisons test *$p < 0.05$, **$p < 0.01$, ***$p < 0.001$, ****$p < 0.0001$ (Speed: ut WT vs ut KO $p = 0.0386$, ut WT vs treh WT $p = 0.8493$, ut WT vs treh KO $p = 0.9714$, ut KO vs treh WT $p = 0.0029$, ut KO vs treh KO $p = 0.0545$, treh WT vs treh KO $p = 0.5258$; Unsupported rearing: ut WT vs ut KO $p = 0.2223$, ut WT vs treh WT $p = 0.9844$, ut WT vs treh KO $p = 0.9177$, ut KO vs treh WT $p = 0.0894$, ut KO vs treh KO $p = 0.0349$, treh WT vs treh KO $p = 0.9918$; Marble: ut WT vs ut KO $p = 0.0032$, ut WT vs treh WT $p = 0.1181$, ut WT vs treh KO $p = 0.1366$, ut KO vs treh WT $p > 0.0001$, ut KO vs treh KO $p = 0.3951$, treh WT vs treh KO $p = 0.0003$). Statistics for the survival curves were performed by log-rank test.

 © The Author(s)
